# Comparing the performance of beamformer algorithms in estimating orientations of neural sources

**DOI:** 10.1016/j.isci.2024.109150

**Published:** 2024-02-06

**Authors:** Yvonne Buschermöhle, Malte B. Höltershinken, Tim Erdbrügger, Jan-Ole Radecke, Andreas Sprenger, Till R. Schneider, Rebekka Lencer, Joachim Gross, Carsten H. Wolters

**Affiliations:** 1 Institute for Biomagnetism and Biosignalanalysis, University of Münster, 48149 Münster, Germany; 2 Otto Creutzfeldt Center for Cognitive and Behavioral Neuroscience, University of Münster, 48149 Münster, Germany; 3 Institute for Analysis and Numerics, University of Münster, 48149 Münster, Germany; 4 Department of Psychiatry and Psychotherapy, University of Lübeck, 23562 Lübeck, Germany; 5 Center of Brain, Behavior and Metabolism, University of Lübeck, 23562 Lübeck, Germany; 6 Department of Neurology, University of Lübeck, 23562 Lübeck, Germany; 7 Institute of Psychology II, University of Lübeck, 23562 Lübeck, Germany; 8 Department of Neurophysiology and Pathophysiology, University Medical Center Hamburg-Eppendorf, 20251 Hamburg, Germany; 9 Institute of Translational Psychiatry, University of Münster, 48149 Münster, Germany

**Keywords:** Neuroscience, Sensory neuroscience

## Abstract

The efficacy of transcranial electric stimulation (tES) to effectively modulate neuronal activity depends critically on the spatial orientation of the targeted neuronal population. Therefore, precise estimation of target orientation is of utmost importance. Different beamforming algorithms provide orientation estimates; however, a systematic analysis of their performance is still lacking. For fixed brain locations, EEG and MEG data from sources with randomized orientations were simulated. The orientation was then estimated (1) with an EEG and (2) with a combined EEG-MEG approach. Three commonly used beamformer algorithms were evaluated with respect to their abilities to estimate the correct orientation: Unit-Gain (UG), Unit-Noise-Gain (UNG), and Array-Gain (AG) beamformer. Performance depends on the signal-to-noise ratios for the modalities and on the chosen beamformer. Overall, the UNG and AG beamformers appear as the most reliable. With increasing noise, the UG estimate converges to a vector determined by the leadfield, thus leading to insufficient orientation estimates.

## Highlights

• UNG and AG beamformers show best orientation estimates in simulation
• UG beamformer orientation estimate subject to noise-dependent bias
• Combined EMEG can outperform pure EEG estimates depending on SNRs
• Results hold in both, simulations and mathematical investigations

## Graphical abstract

**Figure:**
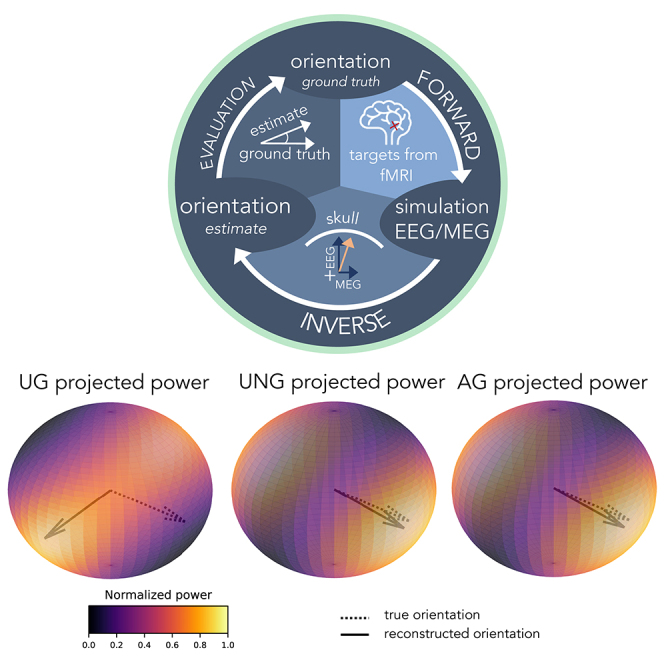

## Introduction

Transcranial electric stimulation (tES) is a promising method to modulate neurons non-invasively. Several studies suggest that the application of tES can reduce symptoms of neurological and mental health disorders such as depression^1^^,^^2^ or epilepsy.^3^^,^^4^^,^^5^ In a conventional tES setup, two electrode patches are placed on the scalp and a small electric current (∼ 0.5–4 mA) is applied in order to modulate membrane polarization.^6^ It is a common approach to place the anode (or cathode) above the target for excitatory (or inhibitory) stimulation.^6^ However, it was shown that this procedure does not maximize the current flow at a given target location, but often in non-target areas.^7^^,^^8^ Furthermore, individual differences in functional targets are neglected.^9^ Importantly, also, the spatial orientation of the neural target population is neglected, which leads to suboptimal stimulation.^8^^,^^9^^,^^10^^,^^11^^,^^12^^,^^13^^,^^14^^,^^15^^,^^16^ Using realistic individual head models, the electric current flow resulting from a certain stimulation electrode setup can be simulated and the tES montage can be optimized.^17^ This allows individually targeted tES considering both target location and orientation to be developed^8^^,^^17^ to maximize the outcome in actual tES applications.^11^^,^^16^^,^^18^ In these approaches multiple (but at least 2) small electrodes are placed on the scalp in a way to maximize the electric current flow in the target area,^8^ while some algorithms additionally minimize it in other areas^15^ while restricting the total sum of injected currents to minimize side effects.^17^ As an electric current flow parallel (or orthogonal) to the neural orientation has shown to maximize (or limit) the stimulation effect, another essential condition for optimizing the stimulation montage is to align the direction of the current flow with the orientation of the stimulation target.^9^^,^^10^^,^^11^^,^^12^^,^^13^^,^^15^^,^^16^^,^^19^ Therefore, knowing the main spatial direction of the targeted neuronal population (i.e., the orientation), is crucial. Determining the orientation can be accomplished in different ways. Neural activity observed in electro- (EEG) and magnetoencephalography (MEG) is generated mainly by the large pyramidal neurons of cortical layer V,^20^ which evoke an electromagnetic field, measurable on the scalp surface with EEG and MEG.^21^^,^^22^^,^^23^ It is often assumed, that these cells are arranged in parallel to each other and oriented orthogonally to the cortex surface,^21^^,^^22^^,^^23^^,^^24^ which offers an approach to determine the orientation anatomically. Results obtained by Bonaiuto et al. suggest, that a linking vector between gray and white matter is the better estimate for the orientation of the target.^25^ tES is closely entangled with bioelectromagnetic data via the Helmholtz reciprocity theorem.^22^^,^^26^^,^^27^ As the target can be modeled as a sum dipole, which in turn can be measured with EEG and MEG, it seems natural to use inverse source analysis based on EEG and MEG data for the target estimation in the context of tES. Only few studies have yet taken into account individual target orientations, estimating it by means of dipole scans^11^ or current density reconstructions.^16^^,^^18^ An alternative inverse method for target reconstruction are beamforming algorithms, which will be evaluated in this study. Beamformers are inverse algorithms designed to only focus on the target activity while spatially filtering out activity originating from interfering sources. Therefore, beamformers are well suited for neural data containing artifacts or weak sources.^28^^,^^29^ While there are studies evaluating the performance of beamformers in source localization^30^^,^^31^ or time series reconstruction,^32^ a systematic evaluation of their performance in orientation reconstruction is still lacking, which leads to the first question, we will address in this study: How well do the beamformers estimate target orientation and which algorithm provides the best orientation estimate? This study attempts to answer this question by testing the performance of three commonly used and implemented beamformer algorithms: Linearly Constrained Minimum Variance (LCMV) beamformers with Unit-Gain (UG),^28^^,^^29^ Unit-Noise-Gain (UNG)^28^^,^^33^ and Array-Gain (AG) constraint.^28^^,^^29^ As the algorithms are implemented and used for both, EEG and MEG data, a second important question is which modality can be used to reliably recreate the underlying orientation. The MEG is known to be insensitive to radially oriented parts of a source’s activity, thus MEG is not recommended to be used to reconstruct source orientation with a radial component.^23^^,^^34^^,^^35^ However, we hypothesize that by combining EEG and MEG (hereafter: EMEG) data for the analysis, the estimation error decreases in comparison to a pure EEG estimate, due to the sensitivity of the MEG to tangential orientation components. This hypothesis is dependent on the accuracy of the underlying head model: only a sufficiently realistic representation of the volume conductor takes the different sensitivity profiles of EEG and MEG adequately well into account to allow a combined use of EEG and MEG, especially with regard to source orientation.^35^^,^^36^^,^^37^^,^^38^ Additionally, the orientation estimation performance shall be evaluated regarding different signal-to-noise-ratios (SNR), which differ in EEG or MEG measurements and are an important parameter in inverse source modeling. In this work, we evaluate beamformer performance for estimating target orientations using simulated data with known source orientation. We choose the posterior visual area V5 and the anterior Frontal Eye Field (FEF) of the right hemisphere as two exemplary realistic simulation targets. We specifically investigate effects of modality (MEG, EEG, and EMEG) and SNR on the three LCMV beamformers (UG, UNG, AG), using a six-compartment finite element head model with anisotropic white matter conductivity, calibrated with regard to individual skull-conductivity.^37^^,^^39^

## Results

In this study, we simulated MEG and EEG data at two realistic target locations (and further sources varying in depth) with different underlying orientations and attempted to reconstruct orientations with the commonly used LCMV beamformer algorithms with (1) UG, (2) UNG and (3) AG constraints. In a first step, we simulated EEG and MEG data with randomly generated target orientations (ground truth) and different noise levels σ, which are a measure of the SNR (for details, see STAR Methods). From MEG data, we only estimated the tangential component of the orientation, for EEG we estimated the full orientation. To obtain a combined EMEG estimate, we recombined the tangential part of the MEG estimate and the radial part of the EEG estimate. For all modalities, we tested all investigated beamformers. The reconstructed orientations were compared to the ground truth orientation. In the second analysis, we repeated this procedure for varying ratios between EEG and MEG noise levels to compare resulting estimation performance in EEG and EMEG. Thirdly, possible orientations are scanned systematically, and these fixed orientations were estimated from simulated EEG and MEG data (for details, see STAR Methods).

### Mathematics summary

Our numerical investigations are complemented by theoretical investigations. In the STAR Methods (section Mathematical Details), we show that for the single source setup used in the numerical simulation, the AG and UNG beamformers have no orientation bias when estimating the complete orientation in the EEG case, as well as when estimating only the tangential component in the MEG case. Furthermore, we show that the UG beamformer exhibits an orientation bias in both the EEG case and the MEG case. Specifically, we present an in-depth investigation of the nature of the UG bias, showing that even in an ideal scenario, for a low SNR the UG reconstruction is essentially determined only by the leadfield and is not expected to give a good estimation of the source orientation. Furthermore, we also show that at a high SNR the EEG UG reconstruction converges to the true source orientation, while the MEG estimation of the tangential component will in general not converge to the true tangential component of the source orientation even at an arbitrarily high SNR. Furthermore, we show that the performance of the UG reconstruction, in addition to suffering from a low SNR, can also be degraded by a high condition number of the leadfield used in the reconstruction. Finally, we show that natural generalizations of the results regarding the bias of the UG beamformer are also valid for arbitrary positive definite noise covariance matrices.

### Random orientations: UNG and AG are more robust toward noise than UG

**Figure 1:**
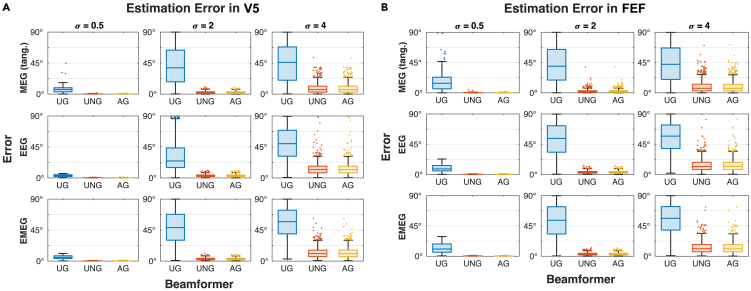
Estimation errors for different orientations in all beamformers (UG, UNG, AG) and modalities (MEG, EEG, EMEG) Angles between estimate and ground truth for 1000 random orientations per condition in V5 (A) and FEF (B) for different beamformers (UG: blue; UNG: orange; AG: yellow). Conditions differ in noise levels (σ=[0.5,2,4]) and modalities (MEG, EEG, combined EMEG). For MEG, the estimation error is evaluated in the tangential plane only. Signal strength is equal in all scenarios. Data are represented as boxplots. See also Figures S2 and S5, Tables S1 and S2.

**Figure 2:**
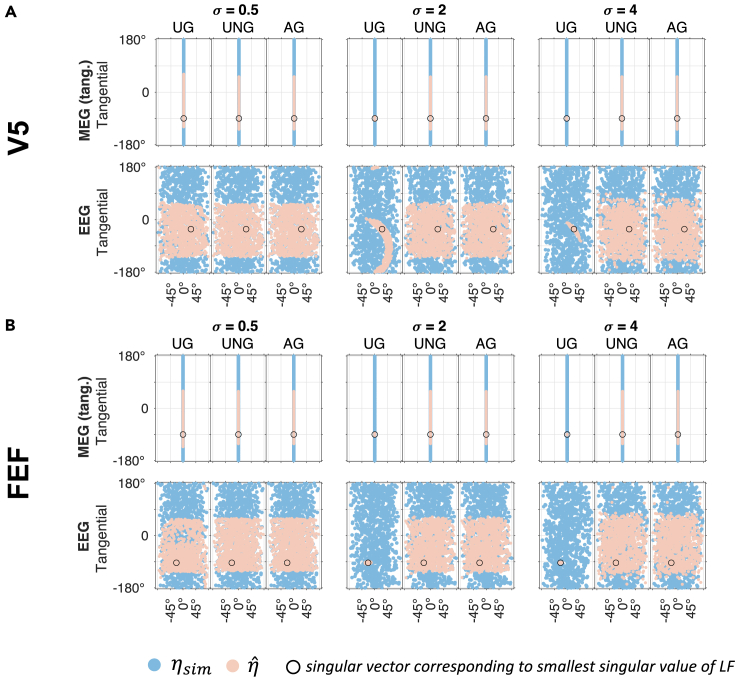
Difference between sets of ground truth orientations and estimated orientations Contrasting the set of simulated orientations $\eta_{\text{sim}}$ (blue) and the reconstructed orientations $\hat{\eta }$ (orange) for varying modalities (MEG and EEG) and noise levels σ in both targets V5 (A) and FEF (B). Coordinates represent the orientations in spherical coordinates (x axis: elevation/radial component; y axis: azimuth/tangential component). While simulated orientations (blue) cover the entire sphere, UG reconstructions converge to a fixed value with increasing noise levels in both targets and modalities. The convergence value is the singular vector corresponding to the smallest singular value of the leadfield (MEG or EEG). In UNG and AG, estimates cover (at least) half the sphere of possible orientations. Since beamformers only reconstruct up to a difference of $180^{\circ }$, this is equivalent to covering the entire sphere of possible orientations and points to no convergence value. Signal strength is equal in all scenarios. See also Figure S6.

The first part of our results presents the orientation estimation error of 1000 randomly generated orientations per noise level σ for each modality with equal signal strengths for all scenarios (Figure 1) (noise level σ therefore represents a measure of the SNR). In each condition, all three beamformer algorithms were evaluated with possible orientation estimation errors between $0^{\circ }$ (perfect estimate) and $90^{\circ }$ (worst estimate; for details, see STAR Methods). First, we consider the effect of noise level σ on the performance at reconstructing the orientation. The results show that for a low noise level (σ=0.5), all three algorithms perform reasonably well in all modalities and both target locations, although the estimation error in UG comprises a larger range of values and is therefore less accurate than the UNG and AG (Figure 1 and Table 1). Increasing the noise to a medium level (σ=2), the error in the UG orientation estimate increases, revealing a high uncertainty in the estimation (Figure 1 and Table 1). In contrast, the errors in the UNG and AG estimates increase as well but are still lower than for UG (Table 1). Although MEG seems to be the most reliable modality to estimate the orientation (Figure 1 and Table 1), it is once again emphasized, that this estimate only considers the tangential components and does therefore not suffice for our purposes. It is only shown for descriptive reasons. This first analysis demonstrates the overall best performance for the UNG and AG beamformers across modalities, noise levels and random orientations. However, a difference between the pure EEG and the combined EMEG estimate cannot be determined properly from these results and is therefore examined in the next section. In general, no systematic differences are found between different targets (Figures 1 and S2, and Table 1). Especially the UNG and AG show the same behavior for targets of different locations and depths (Figures 1 and S2). The UG always performs worse than the other two algorithms, but also shows differences between the targets, which are particularly evident for low noise levels (Figures 1 and S2). As we examined targets differing in depth, we investigated the dependency of the estimation error on the target depth (Figure S3; Table S1). No significant correlations were found in any condition (Table S1). This is congruent with the mathematical analysis (STAR Methods), which shows no dependency of the expected orientation estimate on the target location r. The ratio between the largest and smallest singular values of the leadfield (in the following referred to as the condition number) however, is an important indicator for the convergence of the UG beamformer, as we suggested mathematically in STAR Methods. The results confirm that the median estimation error shows no large differences between targets (error range per condition $<2^{\circ }$) for UNG or AG beamformer (Figure S4). The UG estimation error however shows stronger differences (error range per condition up to $∼15^{\circ }$), which also seem to be connected to the condition number (Figure S4). A statistical analysis confirms this correlation for low (r=0.967, p<0.001, uncorrected for multiple comparisons) and medium noise level (r=0.820, p=0.013, uncorrected for multiple comparisons) (Table S2). For high noise levels, the correlation is not significant (Table S2), however with the limited number of investigated targets, the values must be interpreted with care. We also find a strong correlation between the median estimation error in EEG and the condition number for UNG and AG (Table S2). Additional insight on the insufficient orientation estimation of the UG reveals the contrast between the set of ground truth orientations ($\eta_{\text{sim}}$) and the set of estimated orientations $\hat{\eta }$ (Figure 2). For increasing values of σ, the UG estimates converge to the singular vector corresponding to the lowest singular value of the leadfield. For UNG and AG, no such convergence is observed. Instead, the sets of estimates fill half the sphere of possible orientations. The reason for the reduction of the set of estimates (half sphere) compared to the set of ground truth orientations (entire sphere) is explained by the beamformer’s insensitivity for antiparallel orientations: two antiparallel ground truth orientations are mapped to the same estimation orientation. The set of estimates covers half of the possible orientations, which is equivalent to cover all possible orientations and therefore no systematic preference for the orientation is deduced from these results in AG and UNG. This finding also applies to extremely high noise levels ($\sigma =\left[10^{2},10^{3},10^{4}\right]$), where the estimate errors for UNG and AG are equally high as for UG (Figure S6), showing that there is no convergence for UNG or AG estimates.

**Table 1:** Confidence intervals of orientation estimation errors for random orientations

	σ	V5			FEF		
		UG	UNG	AG	UG	UNG	AG
MEG	0.5	[5.98; 6.44]	[0.23; 0.25]	[0.22; 0.25]	[14.21; 15.52]	[0.24; 0.28]	[0.23; 0.26]
	2	[39.37; 42.68]	[2.15; 2.37]	[2.13; 2.34]	[40.31; 43.59]	[2.20; 2.52]	[2.16; 2.47]
	4	[43.25; 46.54]	[7.93; 8.84]	[7.83; 8.73]	[42.07; 45.33]	[8.65; 9.73]	[8.55; 9.62]
EEG	0.5	[3.15; 3.35]	[0.32; 0.34]	[0.32; 0.34]	[9.18; 9.84]	[0.34; 0.37]	[0.34; 0.36]
	2	[30.30; 32.99]	[3.19; 3.40]	[3.18; 3.39]	[51.23; 54.09]	[3.38; 3.63]	[3.37; 3.62]
	4	[48.29; 51.11]	[12.78; 13.84]	[12.74; 13.80]	[53.70; 56.47]	[13.32; 14.46]	[13.27; 14.40]
EMEG	0.5	[5.46; 5.78]	[0.30; 0.32]	[0.30; 0.32]	[11.86; 12.79]	[0.32; 0.34]	[0.32; 0.34]
	2	[47.68; 50.47]	[2.95; 3.16]	[2.93; 3.14]	[51.28; 54.15]	[3.14; 3.39]	[3.12; 3.36]
	4	[53.94; 56.66]	[11.59; 12.54]	[11.51; 12.46]	[53.71; 56.49]	[12.28; 13.37]	[12.19; 13.27]

95% confidence intervals of the orientation estimation error of 1000 randomly generated orientations for different modalities (MEG, EEG, EMEG), noise levels, beamformers (UG, UNG, AG) and targets (V5, FEF). Values are indicated in degrees. FEF: Frontal Eye Field; UG: Unit-Gain; UNG: Unit-Noise-Gain; AG: Array-Gain; MEG: Magnetoencephalography; EEG: Electroencephalography; EMEG: combined EEG and MEG; σ: Noise level.

### MEG- and EEG-specific noise levels influence the orientation estimation error

**Figure 3:**
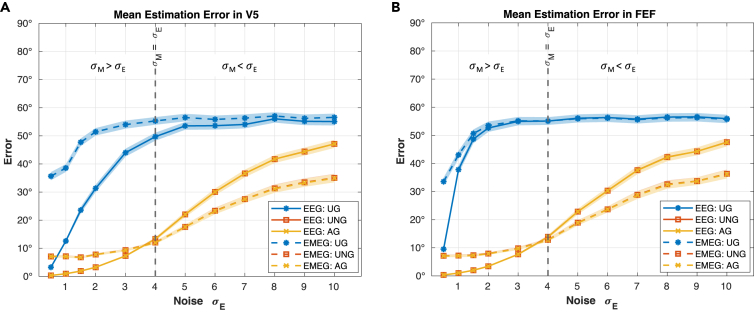
Mean estimation errors for varying $\sigma_{E}$ 95% confidence intervals of mean orientation estimation error for increasing noise levels in simulated EEG data for all three beamformer algorithms (UG: blue; UNG: orange; AG: yellow) and the modalities EEG (solid lines) and EMEG (dashed lines) in V5 (A) and FEF (B). The noise level in MEG data is kept constant ($\sigma_{M}=4$). Signal strength is equal in all scenarios. See also Tables S3 and S4.

We expect the orientation estimate to be more accurate using combined EMEG data than only EEG data. In this context, the noise level of the simulated data, especially the ratio between EEG noise level $\sigma_{E}$ and MEG noise level $\sigma_{M}$, most likely plays an important role. In the previous result section, the noise levels were kept equal between MEG and EEG ($\sigma_{E}=\sigma_{M}$), where the different scaling and units of EEG and MEG is accounted for as described in Equation 11. However, the assumption of equal noise levels between modalities cannot be generalized and, in realistic cases the noise levels will differ. Figure 3 therefore shows the estimation error depending on increasing $\sigma_{E}$ (range: $\sigma_{E}$ = 0.5 to 10), with $\sigma_{M}$ kept constant ($\sigma_{M}=4$). Following the UNG and AG performance curves from low to high values of $\sigma_{E}$, one discovers that the EEG outperforms the EMEG for values of $\sigma_{E}<\sigma_{M}$, while it is vice versa for $\sigma_{E}>\sigma_{M}$. In cases of equal noise levels, the EMEG performs slightly better than the pure EEG estimate (Figure 3; Tables S3 and S4). The results are valid for both, V5 and FEF. In accordance with the results of the previous section, the UG produces the highest estimation error among the three algorithms in all modalities (Figure 3; Tables S3 and S4). While for UG in V5, the combined EMEG is the worse estimate compared to the EEG estimate even for relatively high $\sigma_{E}$ (Figure 3; Table S3), in FEF they perform a lot more similar (Figure 3; Table S4). Overall, however, the UG algorithm performs much worse than the other two algorithms (Figure 3, Tables S3 and S4).

### Fixed orientations: No orientation preference for UNG and AG

**Figure 4:**
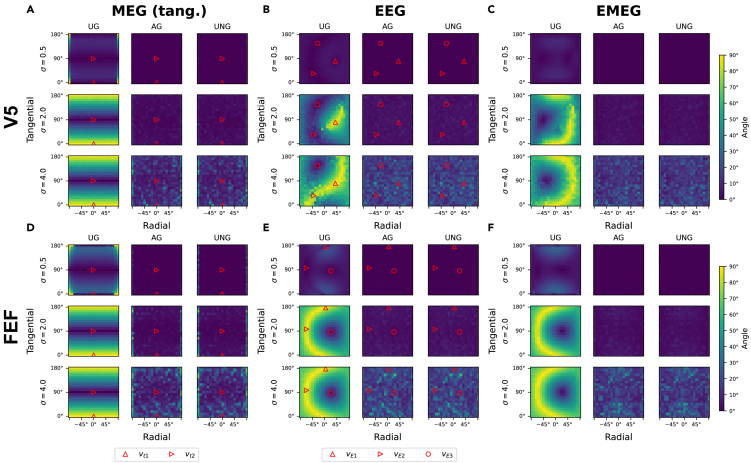
Median estimation errors for fixed orientations Median orientation estimation error depending on the orientation, presented in spherical coordinates (x axis: elevation/radial component; y axis: azimuth/tangential component) for different modalities (MEG: A, D; EEG: B, E; EMEG: C, F), noise levels (0.5, 2, 4) and targets (V5: A–C; FEF: D–F). Assigned colors represent the orientation estimation error on a scale of $0^{\circ }$ to $90^{\circ }$. The red markers represent the singular vectors of the respective leadfield (in MEG and EEG). Signal strength is equal in all scenarios.

In the last two paragraphs, we have seen general estimation errors for the reconstruction of random orientations, but the question remains, if the estimation error depends on the underlying ground truth orientation. To answer this question, the estimation error was evaluated for 400 fixed orientations, which were distributed over the sphere of possible orientations a target can have (Azimuth/tangential plane: from $0^{\circ }$ to $180^{\circ }$ Elevation/radial direction: $-90^{\circ }$ to $90^{\circ }$; each in steps of $9^{\circ }$). For both targets and all modalities, again three values for the noise level (σ=[0.5,2,4]) were analyzed for all three beamformers. What stands out are the results from the UG algorithm, showing a strong dependency of estimation error on orientation which grows with increasing noise level: For the lowest noise level (σ=0.5), the median estimation error is comparatively low in all tested orientations (Figure 4). Increasing the noise to a medium level (σ=2), the estimation error for certain orientations increases to $90^{\circ }$ (Figure 4), meaning that these orientations are estimated consistently wrong. For the highest noise (σ=4), the number of orientations that is estimated inaccurately is increased in EEG and EMEG, while in MEG no differences are noticeable (Figure 4). The estimation error in UG is always lowest for the singular vector corresponding to the lowest singular value of the leadfield (Figure 4). In contrast, the estimation error is highest for the singular vector corresponding to the highest singular value and even reaches maximal values in high noise conditions (Figure 4). Apart from the different singular vectors, there are no systematic differences between the results of V5 and FEF. For the UNG and AG, the estimation errors show no systematic dependency on source orientation in any condition but seem randomly distributed over orientations. Consistent with the previously presented results, the estimation error is lowest for the UNG and AG beamformers (Figure 4). Estimation errors for EMEG are slightly lower than for EEG in both algorithms. The results between FEF and V5 do not differ for UNG and AG (Figure 4).

## Discussion

In this study we compared the performance of reconstructing the orientation with three different versions of the LCMV beamformer, namely the UG,^29^ the UNG^28^^,^^33^ and the AG beamformer.^28^^,^^29^ The results (Figure 1) show, that the UNG and AG beamformers lead to the most accurate estimates of a source’s orientation. Concerning the modality (EEG, MEG, EMEG), we demonstrated that the combined EMEG analysis is beneficial, especially when the noise level in EEG is higher than in MEG data (Figure 3). Furthermore, we found, that the performance of the UG is generally poor as the orientation estimation converges to a value determined by the leadfield, not by the data (Figures 2 and 4). For UNG or AG no dependence of the orientation estimate on the ground truth orientation and no convergence was observed (Figures 2, 4, and S6). The results offer a substantial benefit to make an informed decision about which beamformer algorithm to use when interested in the source orientation of a given neural generator. Thus, especially the UNG and AG beamformers offer the possibility to compute a reasonably accurate estimate of the orientation, even in cases with low SNR or where artifacts indicate the necessity of a spatial filter approach. Concerning the choice of beamformers we suggest, that the UG beamformer provides insufficient orientation estimates and should be avoided when estimating target orientations. According to our results the estimate converges to an orientation, which is determined by the leadfield rather than being determined by the EEG or MEG data. Importantly, the convergence can also be shown mathematically: For increasing noise, the expectation value of the reconstructed orientation shifts from the true orientation or one close to it (for σ→0) to the singular vector corresponding to the smallest singular value of the leadfield matrix (for σ→∞) as presented in detail in the STAR Methods. In this simulation we additionally show that the UG estimation of orientations does not only converge for infinite, but also for finite noise levels, for which the AG and UNG still work well. These results are congruent with previous findings by Neugebauer et al. (2017),^31^ that imply a bad estimation of the orientation for the UG and point out the resulting loss of localization accuracy. Similar results were also reported by Johnson et al. (2011),^40^ who found strong radial components when reconstructing tangentially oriented sources. They concluded that the scalar UG beamformer is not suited to reconstruct non-radial source orientations. Our study adds to these findings the insight, that the problem we face with the UG beamformer, is rooted in the algorithm itself. Especially we want to stress, that the associated problems do not result from the weak radial component in MEG, since we observe the same issues in (1) EEG data and (2) MEG data reduced to the tangential plane and (3) offer a mathematical proof for the UG convergence (STAR Methods). Since there is no ground truth for comparison in realistic data, the observed convergence of the UG orientation estimates emphasizes the importance of our investigation using a simulation approach. The question therefore remains as to whether there is a difference between the beamformer performances in different targets. For UNG and AG, there is no preference for a certain target in the present analyses (Figures 1, 3, 4, and S2). This is in accordance with our mathematical findings, as the expected orientation estimate does not depend on the target location. However, one should keep in mind, that activity from deeper sources tends to have a lower SNR and we therefore expect the estimate of deeper (or other weak) sources to be less accurate than the estimate of more superficial sources. For UG, we see a difference between the estimation errors in the investigated targets: especially for small noise levels, we see an overall better estimation of the orientation in V5 when compared to FEF (Figures 1, 3, 4, and S2). According to our analysis, this seems to be the result of a dependency of the estimation error on the condition number. This correlation can be explained with the convergence of the UG beamformer. In the mathematical analysis, we found the condition number to be an indicator of the convergence speed. What we found in the simulation results represents the mathematical findings: the larger the condition number of a target leadfield, the higher the median estimation error for the same noise level. For the lowest noise levels, the UG estimates are still comparatively far away from the convergence orientation. With increasing noise levels, the correlation decreases as the UG estimates reach the convergence orientation, and the error does not increase anymore – hence a lower correlation with the condition number (Figure S4; Table S2). The correlations between the condition number and median estimation error of the UNG and AG beamformer in EEG cannot be explained mathematically. However, since compared to the UG beamformer the median error of UNG and AG barely differs between targets (Figure S4), these correlations are not meaningful in this context. To confirm these results statistically profound, we suggest a whole-brain analysis, which is beyond the scope of our investigations. For FEF, the UG estimation errors hardly differ between EEG and EMEG, while in V5, the EMEG estimate performs even worse than the pure EEG estimate. This discrepancy can be explained by the specific singular vectors of FEF and V5. The singular vectors corresponding to the lowest singular values in V5 are very different in EEG and MEG, while they are rather close in FEF (Figure 3). Since the UG estimates always converge to these singular vectors, it makes sense that the EEG and EMEG estimates are closer to each other in FEF than they are in V5. As discussed above however, UG estimates for sufficient noise levels (here: σ≳2) do not reflect the actual orientation, but mainly characteristics of the leadfields. Consequently, the reduced performance of EMEG compared to EEG (Figures 1 and 3, Tables S3 and S4) should not be taken as an argument against EMEG. In general, one can conclude that the actual noise levels in the EEG and MEG data are decisive when it comes to the performance of the modalities (Figure 3). If the noise level is known, comparing the corresponding parameters with the presented results might help deciding, which modality should be preferred. However, estimating $\sigma_{E}$ and $\sigma_{M}$ comes with its difficulties. Previous research has shown that the noise level of a source depends on its location depth and orientation.^35^^,^^41^ As the MEG is more sensitive to tangential sources, it is therefore more sensitive to superficial than to deep sources since the latter are more similar to radial sources. Hence, one can expect that MEG signals of subcortical or deep cortical sources contain stronger noise levels than EEG signals. Another important aspect is the volume conductor model of the head as it plays a vital role in this context. For our realistic six compartment headmodel, most cortical areas show a lower noise level in MEG data than in EEG data^35^ and therefore the combined EMEG analysis is recommended for those sources. In summary, our study clearly identified the UNG and AG beamformers as the most reliable choice, when it comes to reconstructing the source orientation (regardless of modality). The UG beamformer should be avoided, since it does not reconstruct the orientation, but leadfield parameters. The choice of modality depends on the noise level of the data. Since most cortical sources have a higher SNR in MEG than in EEG data, using the combined analysis will be beneficial in most cases.

### Limitations of the study

As we aim to present a mathematically controllable simulation, the main limitation of this simulation study is the assumption of Gaussian Noise. Note however, that for a sufficiently good estimate of the noise covariance matrix, prewhitening of the data leads to a covariance matrix similar to the one derived from the investigated Gaussian Noise. Our simulated data contains only one active source to whose signal Gaussian Noise is added. Real data will contain multiple active sources, which are potentially correlated. While especially correlated sources are known to limit beamformer performances (e.g., in localization accuracy),^29^ this is a very important topic to be investigated in future research, as it goes beyond the scope of this study. Additionally, a realistic combination of values for the noise levels $\sigma_{E}$ and $\sigma_{M}$ is difficult to estimate and the utilized values should therefore be interpreted with care. Furthermore, although orientation estimates were assessed for two realistic cortical target locations and 6 additional target locations varying in depth, future studies need to investigate if the observed robust (insufficient) orientation of the UNG and AG (UG) holds true for various other cortical locations. Finally, our results hold only for single active targets, with *a priori* known location and leadfield. In many applications, researchers will estimate the location and orientation in the same process, using numerically computed leadfields. Orientation estimation errors will then be entangled with the localization estimation error and the one in the leadfield. Our study provides important insight into the estimation of a target orientation. However, it also raises open questions, which should be addressed in the future. The results should be validated in real data applications, for example by comparing tES effects for montages optimized based on our estimated beamformer orientations with effects for montages based on other orientations. Comparisons to other orientation estimation methods (e.g., in dipole fits for evoked potentials or fields), could additionally give insight into the transferability of our results to real data. Further, another approach to validate our results further might compare the estimated orientations to anatomically determined orientations as for example in Bonaiuto et al. (2020),^25^ although the estimated orientation from EEG/EMEG summed electromagnetic activity data might not directly correspond to the main anatomical orientation of cortical pyramidal cells.

## STAR★Methods

### Key resources table

**Table:** 

REAGENT or RESOURCE	SOURCE	IDENTIFIER
**Deposited data**		

EEG + MEG leadfields of targets V5 and FEF	This study	https://doi.org/10.5281/zenodo.10492310

**Software and algorithms**		

Python code for present simulation	This study	https://doi.org/10.5281/zenodo.10492310
FieldTrip	Oostenveld et al., 2010	fieldtriptoolbox.org; RRID: SCR_004849
Matlab R2022b	The Mathworks	mathworks.com; RRID: SCR_001622

### Resource availability

#### Lead contact

Further information and requests for resources should be directed to and will be fulfilled by the lead contact, Yvonne Buschermöhle (yvonne.buschermoehle@uni-muenster.de).

#### Materials availability

This study did not generate new unique reagents.

#### Data and code availability

• EEG and MEG leadfields for the targets V5 and FEF have been deposited on zenodo and are publicly available as of the date of publication. The access link is listed in the key resources table.
• All original code has been deposited on zenodo and is publicly available as of the date of publication. The access link is listed in the key resources table.
• Any additional information required to reanalyze the data reported in this paper is available from the lead contact upon request.

### Experimental model and study participant details

We collected data for one native German speaking participant (male; 27 years old) with normal vision, who provided written informed consent. The experimental design was approved by the local ethics committees of the Universities of Münster (Ref: 2015-263-f-S) and Lübeck (Ref: 20-459).

### Method details

The main goal of this study was to evaluate the accuracy to estimate source orientations using different beamformer algorithms. Structural Magnetic Resonance Imaging (MRI) data was recorded to define a realistic six compartment finite-element volume conductor head model for N=1 subject, including white matter anisotropy and skull conductivity calibration. Two realistic cortical target locations of the oculomotor brain network were defined based on functional MRI (fMRI) data of the same subject during smooth pursuit eye movements. For those two cortical locations, three beamformers (UG, UNG, AG) were compared with respect to their ability to reconstruct known orientations for simulated EEG and MEG data. Furthermore, orientation estimates for EEG only, combined EMEG data and tangential orientations in the MEG were assessed.

#### Data acquisition (fMRI and MEG/EEG)

MRI data were recorded using a 3-T Siemens Magnetom Skyra scanner (Siemens, Germany) and a 64-channel head coil. Structural T1 and T2, as well as diffusion weighted images were acquired. Furthermore, horizontal smooth pursuit eye movements together with blood oxygen level dependent (BOLD) activity were recorded. The participant performed smooth pursuit foveating a red dot (size 0.5°) in the framework of a continuous triangle pursuit task (four blocks, 18.7°/s ramp velocity, ±15° amplitude). Each block was preceded by a fixation interval with the red dot presented at the center of the screen (12 s duration; NordicNeuroLab, Norway). Eye movements were recorded using a video-based Eyelink 1000Plus eyetracker system (1000 Hz sampling rate; SR Research Ltd., Canada).

Simultaneous MEG (275 axial gradiometers; OMEGA2005, VSM MedTech Ltd., Canada) and EEG (60 electrodes; EASYCAP GmbH, Herrsching, Germany) data were collected during a median nerve stimulation at the participant’s left wrist. The stimulation comprised 1932 monophasic square-wave electrical impulses of 0.5 ms width and a stimulus onset asynchrony varying between 0.35 ms and 0.45 ms. For the participant’s convenience, the amplitude was chosen as low as possible but such that the thumb moved involuntarily.

#### Target location definition and leadfield computation

EEG and MEG data from visual stimulation often contain eye movement artefacts, which can be difficult to remove. We therefore exemplarily defined the visual area V5 and the FEF (both in right hemisphere) as regions of interest of the oculomotor brain network.^42^^,^^43^^,^^44^^,^^45^ The corresponding target locations were drawn from fMRI data. Functional images were corrected for slice-timing, motion corrected, bias-corrected, spatially normalized to a standard template (Montreal Neurological Institute, MNI) and spatially smoothed. Functional MRI time series were modeled using a general linear model (GLM) including regressors for the start of each stimulation trial convolved with the canonical hemodynamic response function implemented in SPM12. The six motion parameters were defined as covariates in the GLM. Individual locations of right visual area V5 (MNI x/y/z = 48/-61/-7) and right Frontal Eye Field (FEF; MNI x/y/z = 36/-1/44) were determined based on statistical maps showing significantly increased BOLD activity during triangle pursuit blocks in contrast to fixation intervals (FWE-corrected). The locations were defined as the local minima near putative V5 and FEF regions that have previously been shown to signal brain activity during smooth pursuit eye movements in a sample of healthy individuals.^43^^,^^45^ For the subsequent analysis, the identified positions of individual V5 and FEF were re-transformed to native space coordinates using the inverse deformation field maps resulting in our final targets $r_{\;V5}$ and $r_{\;\text{FEF}}$. Six further sources close to the target locations $r_{\;V5}$ and $r_{\;\text{FEF}}$ (three each), but varying in depth, were identified and analyzed (Figure S1).

Realistic 6-compartment (white matter, gray matter, cerebrospinal fluid (CSF), spongiosa, compacta, scalp) head models including anisotropic white matter were created from the aforementioned T1-/T2 and DTI sequences. Using the EEG/MEG data from the median nerve stimulation, the skull conductivity was estimated such that the source reconstructions of the P20/N20 somatosensory component are coherent between EEG and MEG data.^39^ A geometry adapted hexahedral mesh^46^ was created from the segmentation and leadfields for MEG ($L_{M}\left(r\right)$) and EEG ($L_{E}\left(r\right)$) were computed using a first order finite element method. The St. Venant model was used to simulate dipolar neural sources as implemented into DUNEuro.^47^

Parts of the analyses were also performed on sources close to $r_{\;V5}$ and $r_{\;\text{FEF}}$ varying in depth. Based on the realistic head model, we computed the connecting vector c between $r_{\;V5}$ (or $r_{\;\text{FEF}}$) and the closest point of the segmented skull surface and defined sources with different distances (0.5c, 1.5c, 2.5c) to the skull surface. The closest points on the source grid to these target locations were determined and the final distance from the skull was computed for each grid point.

#### Scalar LCMV beamformers and optimal orientations

For an EEG or MEG measurement with $N_{\text{ch}}$ channels, let $L\left(r\right)\in R^{N_{\text{ch}}\times 3}$ be the solution of the forward problem as described above, s(r,t) the source magnitude over time, $\eta \left(r\right)\in R^{3}$ the source orientation and $n\left(t\right)\in R^{N_{\text{ch}}}$ the noise strength. Then the data $d\left(t\right)\in R^{N_{\text{ch}}}$ can be described as

$$d\left(t\right)=\sum_{i=1}^{N}L\left(r_{i}\right)\eta \left(r_{i}\right)s\left(r_{i},t\right)+n\left(t\right)=\sum_{i=1}^{N}l_{\eta }\left(r_{i}\right)s\left(r_{i},t\right)+n\left(t\right)\text{,}$$

where $l_{\eta }\left(r\right)=L\left(r\right)\eta \left(r\right)$ denotes the gain vector in the direction η(r). If we define the source at location $r_{j}$ as the target of interest, we can rewrite the data as the sum of the target signal ($1^{\text{st}}$ addend), signals from other sources ($2^{\text{nd}}$ addend) and those from outside the source space ($3^{\text{rd}}$ addend) as

$$d\left(t\right)=l_{\eta }\left(r_{j}\right)s\left(r_{j},t\right)+\sum_{i\neq j}^{N}l_{\eta }\left(r_{i}\right)s\left(r_{i},t\right)+n\left(t\right)\text{.}$$

To compute an estimate $\hat{s}\left(r_{j},t\right)$ of the signal $s\left(r_{j},t\right)$, we want to find a spatial filter $w\left(r_{j}\right)$ that fulfills

$$\hat{s}\left(r_{j},t\right)=w^{⊺}\left(r_{j}\right)d\left(t\right)\text{.}$$

The expected power of the time discrete signal is approximated by

$$\left\langle {\left|\hat{s}\left(r_{j},t\right)\right|}^{2}\right\rangle =\left\langle {\left|w^{⊺}\left(r_{j}\right)d\left(t\right)\right|}^{2}\right\rangle =\left\langle {\left|w^{⊺}\left(r_{j}\right)\left(l_{\eta }\left(r_{j}\right)s\left(r_{j},t\right)+\sum_{i\neq j}^{N}l_{\eta }\left(r_{i}\right)s\left(r_{i},t\right)+n\left(t\right)\right)\right|}^{2}\right\rangle \text{,}$$

where ⟨·⟩ denotes the time average.

An ideal filter would let the signal produced at the target location $r_{j}$ pass, while it suppresses the signals produced at other sources or outside the source space. In realistic scenarios, it is not possible to wholly suppress the signals generated by other sources. However, they can be partially suppressed by minimizing the power under a constraint, which ensures that the signal of interest is passed. Using the definition $R:=\left\langle d\left(t\right)d{\left(t\right)}^{⊺}\right\rangle$ this is expressed as

$$w\left(r,\eta \left(r\right)\right)=\underset{w\left(r,\eta \right)}{\mathrm{argmin}}\left\langle {\left|w^{⊺}\left(r,\eta \left(r\right)\right)d\left(t\right)\right|}^{2}\right\rangle =\underset{w\left(r,\eta \right)}{\mathrm{argmin}}\left(w^{⊺}\left(r,\eta \left(r\right)\right)\left\langle d\left(t\right)d{\left(t\right)}^{⊺}\right\rangle w\left(r,\eta \left(r\right)\right)\right)=\underset{w\left(r,\eta \right)}{\mathrm{argmin}}\left(w^{⊺}\left(r,\eta \left(r\right)\right)Rw\left(r,\eta \left(r\right)\right)\right)\text{,}$$

subject to

(Equation 1) $$w_{\text{UG}}^{⊺}\left(r,\eta \left(r\right)\right)l_{\eta }\left(r\right)=1\text{,}$$

or

(Equation 2) $$w_{\text{UNG}}^{⊺}\left(r,\eta \left(r\right)\right)l_{\eta }\left(r\right)=\tau ,w_{\text{UNG}}^{⊺}\left(r,\eta \left(r\right)\right)w_{\text{UNG}}\left(r,\eta \left(r\right)\right)=1\text{,}$$

or

(Equation 3) $$w_{\text{AG}}^{⊺}\left(r,\eta \left(r\right)\right)l_{\eta }\left(r\right)=∥l_{\eta }\left(r\right)∥\text{.}$$

The constraints distinguish the three different beamformers. The UG^29^ constraint ensures, that signal from location r is passed in a way that is produces a signal of strength 1 on sensor level. This leads to a depth bias since deep sources with weaker leadfields need to have higher power to evoke the same signal. The UNG^28^^,^^29^^,^^33^ and the AG^28^^,^^29^ constraints include different normalization strategies to account for this depth bias: The UNG normalizes by the norm of the filter (Equation 2) and the AG normalizes the leadfield (Equation 3), which will lead to different estimation measures of the orientation (cf. Equations 7, 8, and 9).

In practice, the second moment matrix R is estimated from the measured data and is customarily called the covariance matrix (as it is equivalent to it for zero mean data, which is often the case).

Solving the constrained minimization problems with Lagrange multipliers, the filters are computed as (for explicit derivations see Sekihara & Nagarajan (2008),^28^ chapter 4.)

(Equation 4) $$w_{\text{UG}}\left(r,\eta \left(r\right)\right)=\frac{R^{-1}l_{\eta }\left(r\right)}{l_{\eta }\left(r\right)R^{-1}l_{\eta }\left(r\right)}\text{,}$$

(Equation 5) $$w_{\text{UNG}}\left(r,\eta \left(r\right)\right)=\frac{R^{-1}l_{\eta }\left(r\right)}{\sqrt{l_{\eta }\left(r\right)R^{-2}l_{\eta }\left(r\right)}}\text{,}$$

(Equation 6) $$w_{\text{AG}}\left(r,\eta \left(r\right)\right)=\frac{R^{-1}{\tilde{l}}_{\eta }\left(r\right)}{{\tilde{l}}_{\eta }\left(r\right)R^{-1}{\tilde{l}}_{\eta }\left(r\right)}\text{,}$$

where ${\tilde{l}}_{\eta }\left(r\right)=\frac{l_{\eta }\left(r\right)}{∥l_{\eta }\left(r\right)∥}$. As can be seen, the filters still depend on the source orientation η. To estimate the orientation from the data, we assume that the optimal orientation maximizes the resulting signal power. We therefore maximize the power with respect to η, using the filters computed in Equations 4, 5, and 6 via

$$\hat{\eta }\left(r_{j}\right)=\underset{\eta \left(r\right)}{\mathrm{argmax}}\left\langle {\left|\hat{s}\left(r_{j},t\right)\right|}^{2}\right\rangle$$

subject to constraints in Equations 1, 2, and 3. Using the Rayleigh-Ritz formula, the optimization problems can be rewritten in terms of generalized eigenvalue problems as (for explicit derivations see Sekihara & Nagarajan (2008),^28^ section 13.3.)

(Equation 7) $${\hat{\eta }}_{\text{UG}}\left(r\right)=\nu_{\min}\left\{L{\left(r\right)}^{⊺}R^{-1}L\left(r\right)\right\}\text{,}$$

(Equation 8) $${\hat{\eta }}_{\text{UNG}}\left(r\right)=\nu_{\min}\left\{L{\left(r\right)}^{⊺}R^{-2}L\left(r\right),L{\left(r\right)}^{⊺}R^{-1}L\left(r\right)\right\}\text{,}$$

(Equation 9) $${\hat{\eta }}_{\text{AG}}\left(r\right)=\nu_{\min}\left\{L{\left(r\right)}^{⊺}R^{-1}L\left(r\right),L{\left(r\right)}^{⊺}L\left(r\right)\right\}\text{,}$$

where $\nu_{\min}\left(A,B\right)=\nu_{\min}\left(B^{-1}A\right)$ is the eigenvector corresponding to the lowest eigenvalue $\lambda_{\min}$ solving the eigenvalue problem ${A\nu }_{\min}=\lambda_{\min}{B\nu }_{\min}$.

#### Source activity and simulated data

The simulated EEG and MEG data are based on the leadfields and targets above. The data were considered as the EEG/ MEG signal evoked by a source at the target superposed with noise to make the data more realistic. The unitless simulated sensor data ${\bar{d}}_{\text{sim}}\left(t\right)\in R^{N_{\text{ch}}}$, created by a source at r with orientation $\eta_{\text{sim}}\in R^{3}$ is defined in Equation 10. The signal is defined in a way to obtain the same signal strength for each source, independent of the source location.

(Equation 10) $${\bar{d}}_{\text{sim}}\left(t\right)=\frac{L\left(r\right)\eta_{\text{sim}}}{∥L\left(r\right)\eta_{\text{sim}}∥}\cdot s_{\text{sim}}\left(t\right)$$

Here, $s_{\text{sim}}\left(t\right)$ describes the simulated source magnitude at time *t* and is defined as

$$s_{\text{sim}}\left(t\right)=\sin\left(2\pi f_{q}t\right)$$

with the source oscillation frequency $f_{q}$ being chosen as 20 Hz. In this simulation, time *t* comprised 60 s, and the sampling frequency was chosen as 600 Hz resulting in a total of T=36000 simulated time points. Temporally and spatially independent Gaussian noise $n_{\text{sim}}\left(t\right)\in R^{N_{\text{ch}}}$, scaled to standard deviation σ, was added to the signal at each time sample. The simulated data vector $d_{\text{sim}}\left(t\right)$ at time t was therefore defined by

(Equation 11) $$d_{\text{sim}}\left(t\right)={\bar{d}}_{\text{sim}}\left(t\right)+\sigma n_{\text{sim}}\left(t\right)\text{.}$$

As the signal ${\bar{d}}_{\text{sim}}$ contains the same power in all investigated scenarios, the parameter σ is a measure of the ratio between signal and noise power. In the following, we refer to σ as the noise level.

The second moment matrix is estimated by

$$R=\frac{1}{T}\sum_{i=1}^{T}d_{\text{sim}}\left(t_{i}\right)d_{\text{sim}}^{⊺}\left(t_{i}\right)\text{.}$$

#### Target specific coordinates $v_{t_{1}}$-$v_{t_{2}}$-$v_{r}$

It is well-known that MEG is relatively insensitive to the radial component of source orientation.^48^ Since we aimed to observe the effect of modality (MEG, EEG, EMEG) on accuracy of estimated source orientation we estimated source orientation in a target specific coordinate system that aligned to the radial and two tangential orientations ($v_{r}$, $v_{t_{1}}$, and $v_{t_{2}}$). While $v_{r}$ denotes the vector oriented radially towards the skull surface, $v_{t_{1}}$ and $v_{t_{2}}$ are the two perpendicular vectors spanning the plane tangential to the skull surface. The vectors were obtained by performing a Singular Value Decomposition (as implemented in MATLAB) on the MEG target leadfield^49^ as

$$L_{M}\left(r\right)={USV}^{⊺}\text{,}$$

where $U\in R^{N_{\text{ch}}\times N_{\text{ch}}}$, $S\in R^{N_{\text{ch}}\times 3}$ and $V=\left(v_{t_{1}},v_{t_{2}},v_{r}\right)\in R^{3\times 3}$.

#### Estimating orientations

To estimate the source orientation from the simulated data, the covariance matrix R and the respective leadfield L(r) were inserted into Equations 7, 8, and 9. To solve the eigenproblems, the numerical implementation available in Fieldtrip was used.^50^ As even in a realistic six-compartment head model MEG is mainly sensitive to the tangential part of the source activity, only the tangential component of the orientation (in the $v_{t_{1}}$-$v_{t_{2}}$ plane) was estimated from the MEG data. Therefore, the MEG leadfield $L_{M}\left(r\right)$ was reduced to the two tangential dimensions by $L_{M}^{\text{red}}\left(r\right)=L_{M}\left(r\right)\cdot \left(v_{t_{1}},v_{t_{2}}\right)\in R^{N_{\text{ch}}\times 2}$. For EEG, the leadfield $L_{E}\left(r\right)$ was kept complete and the entire 3-dimensional orientation was estimated. In both cases, a regularization of 5% was used.

To obtain a combined EMEG estimate, we exploited the strengths of each modality. As MEG is mainly sensitive to tangential components and additionally barely influenced by conductivity parameters, we estimated the tangential part of the orientation from MEG data. The radial component was then estimated from EEG data, which is sensitive to all source orientations, but also more susceptible to conductivity parameters. The two components were superposed, and the resulting vector was normalized. The estimation error ϵ, the angle between the estimate $\hat{\eta }$ and the ground truth $\eta_{\text{sim}}$, was computed as quality measure

$$\epsilon =\frac{\arccos\left(\left|{\hat{\eta }}^{⊺}\eta_{\text{sim}}\right|\right)}{2\pi }\cdot 360^{\circ }$$

Since beamformer algorithms reconstruct the source power $\left\langle {\left|\hat{s}\left(r_{j},t\right)\right|}^{2}\right\rangle$, orientations enclosing an angle of $180^{\circ }$ are indistinguishable. Hence, we defined the smaller angle between the vectors as the estimation error. It can range between $0^{\circ }$ and $90^{\circ }$ with $0^{\circ }$ meaning the estimate equals the ground truth (parallel or anti-parallel) and $90^{\circ }$ meaning the vectors are orthogonal to each other.

### Quantification and statistical analysis

All simulations were performed at the target locations $r_{\;\text{FEF}}$, $r_{\;V5}$ using the respective data and leadfields.

#### Computational simulations

To obtain an overview of the beamformers‘ performances, 1000 random orientations were generated, and the data was simulated for these orientations with noise levels σ of 0.5, 2, and 4 ($\sigma_{E}=\sigma_{M}$) and with extremely high noise levels ($\sigma =\left[10^{2},10^{3},10^{4}\right]$). The orientations were then estimated from the data and compared to the ground truth orientations resulting in 1000 estimation errors for different orientations. Pearson’s correlation coefficient was computed between the median errror and (1) the source depth and (2) the condition number of the leadfield (ratio of largest and smallest singular value). Furthermore, the set of ground truth orientations was contrasted with the set of estimated orientations.

To answer the question whether the pure EEG or the combined EMEG approach leads to the smallest estimation error, the combination of the noise levels $\sigma_{M}$ and $\sigma_{E}$ was examined. While the noise level of the MEG was kept constant at $\sigma_{M}=4$, the EEG was assigned noise levels between $\sigma_{E}=0.5$ and $\sigma_{E}=10$. The simulation as explained in the previous section was repeated and the mean of the distribution was extracted as a central measure for the estimation error.

Finally, the dependency of the estimation error on the source orientation itself was evaluated. If one considers the target as the center of a unit sphere, each orientation can be considered the unit vector between the center and a point on the spherical surface. To generate orientations, the sphere was scanned systematically using spherical coordinates (Azimuth/tangential plane: from $0^{\circ }$ to $180^{\circ }$ in steps of $9^{\circ }$; Elevation/ radial direction: $-90^{\circ }$ to $90^{\circ }$ in steps of $9^{\circ }$). Using bootstrapping by drawing random data samples with replacement from the full simulated data set, each orientation was estimated 100 times. The median of the 100 estimation errors was then extracted.

#### Mathematical Details

In the Method Details, it was described how beamforming was used to reconstruct the orientation of neural activity. There, three different beamforming strategies, namely UG, AG, and UNG, were presented. We now want to investigate the theoretical reconstruction properties of these strategies in a simple setting generalizing the test setup described in the Method Details, namely a scenario with a single active source in the presence of uncorrelated noise. In this setting, we will mathematically prove that the UG beamformer introduces a noise-dependent bias into the reconstruction of the orientation. In particular, we will show that at small SNRs we cannot expect the orientation reconstructed using the UG beamformer to contain any information about the true source orientation. Furthermore, we will show that AG and UNG beamformers do not introduce such a bias.

##### Preliminaries

We use the notation described in the Method Details. In the following, we will keep the position r fixed and will hence omit it from the notation (i.e., we will write $L_{E}$ instead of $L_{E}\left(r\right)$). Furthermore, we assume that $L_{E}$ has rank 3, and that $L_{M}$ has at least rank 2.

We denote by $L_{M}=U\cdot S\cdot V^{⊺}$ a singular value decomposition of the MEG leadfield, i.e. $U\in R^{N_{\text{ch}}\times N_{\text{ch}}}$, $S\in R^{N_{\text{ch}}\times 3}$, and $V\in R^{3\times 3}$. Let $U=\left(u_{1},u_{2},u_{3},U^{\prime }\right)$, with $U^{\prime }\in R^{N_{\text{ch}}\times \left(N_{\text{ch}}-3\right)}$, $S=\text{diag}\left(s_{1},s_{2},s_{3}\right)$ with $s_{1}\geq s_{2}\geq s_{3}\geq 0$, and $V=\left(v_{1},v_{2},v_{3}\right)$. As described in the Method Details, we reduced the MEG leadfield to the tangential directions. Formally this means that we applied beamforming to the matrix $L_{M}^{\text{red}}$ defined by

$$L_{M}^{\text{red}}=\left(s_{1}\cdot u_{1},s_{2}\cdot u_{2}\right)\in R^{N_{\text{ch}}\times 2}\text{.}$$

Note that then $L_{M}^{\text{red}}\cdot e_{i}=L_{M}\cdot v_{i}$, where 1≤i≤2 and $e_{i}$ denotes the *i*-th unit vector in $R^{2}$. In the following, if $y\in R^{3}$ is an arbitrary vector, we will refer to $y^{t}=\left(v_{1}^{⊺}y,v_{2}^{⊺}y\right)$ as the **tangential component** of y, and to $y^{r}=v_{3}^{⊺}y$ as the **radial component** of y.

We want to model our signal as originating from the fixed position r with the fixed orientation $\eta_{\text{sim}}$. Generalizing from Equation 11, we hence model our signal vector d(t) as

$$d\left(t\right)=L\cdot \left(\eta_{\text{sim}}\cdot q\left(t\right)\right)+n\left(t\right)\text{,}$$

where L is either $L_{E}$ or $L_{M}$, *q* is a scalar function modeling the source magnitude, and n is a noise vector. We assume the components of the noise vector to be mutually uncorrelated, and to be uncorrelated to the source magnitude. Furthermore, we assume the noise components to be identically distributed, and to have zero mean. We call this a **noisy single-source setting**. Using $R=\left\langle d\left(t_{i}\right)d{\left(t_{i}\right)}^{⊺}\right\rangle$, where $d\left(t_{1}\right),\ldots ,d\left(t_{T}\right)$ denote samples of the signal d(t), and letting $Q^{2}:=\left\langle q{\left(t_{i}\right)}^{2}\right\rangle$ and $\sigma^{2}:=\left\langle n_{1}{\left(t_{i}\right)}^{2}\right\rangle$, we then have

(Equation 12) $$R=Q^{2}\left(L\eta_{\text{sim}}\right){\left(L\eta_{\text{sim}}\right)}^{⊺}+\sigma^{2}\;\text{Id,}$$

where **Id** denotes the identity matrix. We call $Q^{2}$ the source power and $\sigma^{2}$ the noise power, and assume $Q^{2}>0$ and $\sigma^{2}>0$. A derivation of Equation 12 can e.g. be found in the book of Sekihara and Nagarajan,^28^ Equation 2.43. Note that since L can be either $L_{E}$ or $L_{M}$, this defines an EEG and a MEG covariance matrix.

We now define that a beamformer **has no EEG orientation bias** if the orientation reconstruction algorithm described in the Method Details, applied to the covariance matrix (Equation 12) and the EEG leadfield $L_{E}$, produces, up to a sign change, the true orientation $\eta_{\text{sim}}$ of the simulated source. Similarly, we define that a beamformer **has no MEG orientation bias** if, as long as the true orientation has a non-zero tangential component, the estimated orientation using the covariance matrix (Equation 12) and the reduced MEG leadfield $L_{M}^{\text{red}}$ is, up to a sign change, given by the orientation defined by the tangential component of the true orientation $\eta_{\text{sim}}$.

Note that even though a beamformer might have no EEG or MEG orientation bias, in practice it can still produce orientations that deviate from the true orientation. This may be because one can typically only estimate the true covariance matrix using the sample covariance matrix. Additionally, the exact leadfield is generally not known but instead approximated using some numerical approach. For a discussion on how beamformers are affected by these types of errors, we again refer to the book of Sekihara and Nagarajan.^28^

In theorem 1 below, we will investigate UG, AG, and UNG beamformers. As described in the Method Details, the reconstructed orientations for these different beamformer approaches are then given by

(Equation 13) $$\eta_{\text{UG}}=\nu_{\min}\left\{L^{⊺}R^{-1}L\right\}$$

(Equation 14) $$\eta_{\text{AG}}=\nu_{\min}\left\{L^{⊺}R^{-1}L,L^{⊺}L\right\}$$

(Equation 15) $$\eta_{\text{UNG}}=\nu_{\min}\left\{L^{⊺}R^{-2}L,L^{⊺}R^{-1}L\right\}$$

where $\nu_{\min}\left(A\right)$ denotes an eigenvector to the smallest eigenvalue of A and $\nu_{\min}\left(A,B\right)$ denotes an eigenvector to the smallest generalized eigenvalue of the pair (A,B), i.e. a vector v with Av=λBv, where λ is minimal. For a derivation of these approaches, we refer to the book of Sekihara and Nagarajan,^28^ sections 4.3 and 13.3.

##### Relationship to previously known results and different beamforming approaches

A number of results regarding the theoretical reconstruction properties of different beamforming approaches have already been derived. Vrba and Robinson^51^ show that for MEG signals in a spherical volume conductor, and assuming a noisy single source setting as described above, the so-called *pseudo-*Z *score* of a position and orientation pair $\left(x_{0},\eta \right)$ peaks for pairs consisting of the true dipole position and an orientation whose tangential component is parallel to the tangential component of the true orientation. This result relies on the fact that for spherical volume conductors, radial dipoles produce no magnetic field outside of the head model, as shown by Sarvas,^48^ and hence does not directly generalize to other volume conductor models. In the following, we will denote the process of using the pseudo-Z score to estimate source parameters as *SAM beamforming*. Using essentially the same argument as Vrba and Robinson,^51^ Sekihara et al.^52^ show that, more generally, an estimation of the orientation based on maximizing the pseudo-Z score, in a noisy single source setting as described above, has no orientation bias when estimating the complete dipole orientation (Note that Sekihara et al.^52^ denote the pseudo-Z score as *output SNR.*). Extending upon this paper, they were able to show that under the assumption of a noisy single source setting AG and UNG beamformers have no location bias.^28^^,^^53^ Furthermore, while they do not explicitly state it, their proof in fact also shows that AG and UNG beamformers have no orientation bias when estimating the complete dipole orientation. Greenblatt et al.^54^ also give proof that in a noisy single source setting the UNG beamformer has no location bias. Another particularly noteworthy contribution is due to Moiseev et al.^55^ In this work, the authors introduce functionals generalizing the pseudo-Z score and the *neural activity index* (NAI, see Van Veen et al.^29^). Moiseev et al. then rigorously prove that maximizing these generalized pseudo-Z- and NAI-functionals gives an unbiased estimation for multiple possibly correlated sources and an arbitrary noise covariance matrix. As a special case, this implies that maximizing the pseudo-Z score, resp. the NAI, provides an unbiased source estimation in a single source setting with an arbitrary noise covariance matrix.

At first sight, this last statement seems like a strong improvement over the results regarding AG and UNG beamformers, since pseudo-Z and NAI allow unbiased source estimation for arbitrary noise covariance matrices, while the corresponding statements for AG and UNG beamformers require a noise covariance matrix of the form $\sigma^{2}\cdot \text{Id}$. But it turns out that, up to a change of variables, AG beamforming is equivalent to NAI beamforming, and UNG beamforming is equivalent to SAM beamforming (i.e. maximizing pseudo-Z). We want to show this. Assume we have data $b=\left(b\left(1\right),\ldots ,b\left(T\right)\right)\in R^{n\times T}$. We then estimate the covariance matrix R as $\frac{1}{T}\cdot b\cdot b^{⊺}$. Denote by N a positive definite matrix to be used as a noise covariance matrix. Now denote by $w\left(x_{0},\eta \right)\in R^{n}$ a spatial filter for the position $x_{0}$ and orientation η, and by $l\left(x_{0},\eta \right)\in R^{n}$ the lead field at position $x_{0}$ and orientation η. The pseudo-Z score is then defined as

$$Z\left(x_{0},\eta \right)=\frac{w{\left(x_{0},\eta \right)}^{⊺}\cdot R\cdot w\left(x_{0},\eta \right)}{w{\left(x_{0},\eta \right)}^{⊺}\cdot N\cdot w\left(x_{0},\eta \right)}\text{,}$$

and, following Moiseev et al.,^55^ the scalar version of the NAI is given by

$$\text{NAI}\left(x_{0},\eta \right)=\frac{l{\left(x_{0},\eta \right)}^{⊺}\cdot N^{-1}\cdot l\left(x_{0},\eta \right)}{l{\left(x_{0},\eta \right)}^{⊺}\cdot R^{-1}\cdot l\left(x_{0},\eta \right)}\text{.}$$

Now let $\tilde{b}=N^{-\frac{1}{2}}\cdot b$ be the pre-whitened signal, and denote by $\tilde{l}\left(x_{0},\eta \right)=N^{-\frac{1}{2}}\cdot l\left(x_{0},\eta \right)$ the corresponding transformed leadfields. If we now compute the covariance matrix of the pre-whitened signal $\tilde{b}$ as $\tilde{R}=\frac{1}{T}\cdot \tilde{b}\cdot {\tilde{b}}^{⊺}$, we see that $\tilde{R}=N^{-\frac{1}{2}}\cdot R\cdot N^{-\frac{1}{2}}$. Denote by ${\tilde{w}}_{\text{AG}}$ the AG beamformer computed using the pre-whitened signal and the transformed leadfield, and denote by ${\tilde{P}}_{\text{AG}}\left(x_{0},\eta \right)={\tilde{w}}_{\text{AG}}{\left(x_{0},\eta \right)}^{⊺}\cdot \tilde{R}\cdot {\tilde{w}}_{\text{AG}}\left(x_{0},\eta \right)$ its projected power. Define ${\tilde{w}}_{\text{UNG}}$ and ${\tilde{P}}_{\text{UNG}}$ analogously. Using the formulas for the AG and UNG beamformers as given in Equations 5 and 6, a straightforward computation shows that

$${\tilde{P}}_{\text{AG}}\left(x_{0},\eta \right)=\text{NAI}\left(x_{0},\eta \right)$$

$${\tilde{P}}_{\text{UNG}}\left(x_{0},\eta \right)=Z\left(x_{0},\eta \right)\text{.}$$

We thus see that searching for the source that maximizes the neural activity index is equivalent to searching for the source maximizing the projected AG power in the pre-whitened signal and searching for the source that maximizes the pseudo-Z score is equivalent to searching for the source maximizing the projected UNG power in the pre-whitened signal. Similarly, performing a change of variables with $N^{-\frac{1}{2}}$ shows that the unbiasedness of SAM beamforming, resp. NAI beamforming, for single sources with an arbitrary noise covariance matrix, is a direct consequence of the unbiasedness of UNG beamforming, resp. AG beamforming, for noise covariance matrices of the form $\sigma^{2}\cdot \text{Id}$. On a further note, we see that statements derived for AG and UNG beamformers under the assumption of noise covariance matrices of the form $\sigma^{2}\cdot \text{Id}$ translate to statements about SAM and NAI beamformers for arbitrary noise covariance matrices, and vice versa. Additionally, as a particular special case note that for noise covariances of the form $\sigma^{2}\cdot \text{Id}$ we have that even without a coordinate change the AG beamformer power is a scalar multiple of the neural activity index, and the UNG beamformer power is a scalar multiple of the pseudo-Z score. Hence in this case we have that the source estimation using AG beamforming is the same as NAI beamforming, and that source estimation using UNG beamforming is the same as SAM beamforming.

We thus see that there are already many results showing the unbiasedness of different versions of AG and UNG beamformers in different settings. Additionally, it is well-known that the UG beamformer is not unbiased, and e.g. introduces a depth bias into the reconstruction (see e.g. Sekihara and Nagarajan,^28^ 5.1.6).

In Theorem 1, we will extend upon the known results, with a particular focus on (un-)biasedness of reconstructed orientations. While the orientations of the different beamformers are defined as maximizers of certain power functionals, it is well-known that for a fixed position, the orientation can also be derived by solving a (generalized) eigenproblem (which is the origin of Equations 13, 14, and 15). While the papers cited above show that AG and UNG beamformers have no EEG orientation bias by investigating these functionals, we will show below how this unbiasedness can be derived in a straightforward algebraic way by computing the eigenvectors of the corresponding generalized eigenproblems. Furthermore, we will also show that the estimation of the tangential component of the orientation using the reduced MEG leadfield $L_{M}^{\text{red}}$ is unbiased for AG and UNG beamformers. Finally, we will give an in-depth discussion on the nature of the bias of the UG beamformer, both for noise covariance matrices of the form $\sigma^{2}\cdot \text{Id}$ and for arbitrary positive definite noise covariance matrices.

##### Investigating the reconstruction properties of beamforming approaches

In the following, if we call a vector v an orientation, we implicitly assume ∥v∥=1. Looking at Equations 13, 14, and 15 , we see that whenever v is a valid reconstructed orientation, then so is −v. The beamformers described thus far can hence not differentiate between orientations of opposing signs. If we want to investigate the limiting behavior of reconstructed orientations, we need to take this non-uniqueness into account. More concretly, if $v_{s}$ is a family of orientations parametrized by some parameter *s*, we say that $v_{s}$ **converges up to sign** to $v_{s_{0}}$ if, as *s* approaches $s_{0}$, we have that $v_{s}$ is eventually arbitrarily close to either $v_{s_{0}}$ or $-v_{s_{0}}$. Furthermore, note that in the following considerations, we keep the source power fixed. Hence increasing or decreasing the noise power corresponds to decreasing or increasing the SNR. We can now state the orientation bias behavior of the different beamforming strategies.

**Theorem 1.** We assume a noisy single-source setting. We then have the following.

(1) The AG and UNG beamformers have no EEG orientation bias and no MEG orientation bias.
(2) The UG beamformer has an orientation bias in the EEG case and the MEG case. More concretely, we have the following.
  (a) In the UG EEG case, as the noise power approaches zero, the reconstructed orientation converges up to sign to the true orientation. As the noise power approaches ∞, the limiting behavior of the reconstructed orientation depends on the relation of the true orientation to the singular vectors of $L_{E}$. More specifically, we have the following.
    i. If the true orientation is not orthogonal to the eigenspace of $L_{E}^{⊺}L_{E}$ corresponding to the smallest singular value of $L_{E}$, the reconstructed orientations converge for σ→∞ up to sign to the orthogonal projection of the true orientation onto this eigenspace.
    ii. If the true orientation is orthogonal to this eigenspace, the reconstructed orientation depends discontinuously on the noise power and is, for sufficiently large noise, given by an arbitrary singular vector of $L_{E}$ corresponding to the smallest singular value.
  (b) In the UG MEG case, the limiting behavior as the noise power approaches ∞ is the same as the behavior in the EEG case, with the true orientation replaced by its tangential projection and $L_{E}$ replaced by $L_{M}^{\text{red}}$. But in contrast to the EEG case, as the noise power approaches zero, the reconstructed orientation does not converge to the tangential component of the true orientation, but instead to some other vector $\tilde{\eta }$, which typically will be quite close to, but different from, the tangential component of $\eta_{\text{sim}}$. We refer to the following proof for the precise form of $\tilde{\eta }$.

**Remark.** We want to add some comments on the UG reconstruction.

(1) In the generic case the eigenspace of $L^{⊺}L$ corresponding to the smallest singular value of L is one-dimensional. In this case, as σ→∞ the UG orientation reconstruction simply converges up to sign to the corresponding singular vector.
(2) It might seem somewhat surprising that the limiting behavior of the UG beamformer as σ→0 is different in the EEG case and the MEG case. The fundamental reason for this is that in the MEG case, the covariance matrix R contains the full leadfield $L_{M}\cdot \eta_{\text{sim}}$, while the reconstruction uses the reduced leadfield $L_{M}^{\text{red}}$. However, note that the MEG signal of a source is generally dominated by its tangential component, which is the reason why the reconstructed orientation $\tilde{\eta }$ will still be quite close to the true orientation. In fact, if $\eta_{\text{sim}}^{t}$ denotes the tangential component of the true orientation, the following proof shows that the difference between $\tilde{\eta }$ and $\eta_{\text{sim}}^{t}$ is caused by the difference between $∥L_{M}\cdot \eta_{\text{sim}}∥$ and $∥L_{M}^{\text{red}}\cdot \eta_{\text{sim}}^{t}∥$.

####### Part 1: Proof that investigating the covariance matrix

As a first step, we need to better understand the covariance matrix R. Letting $x=Q\cdot L\cdot \eta_{\text{sim}}$, we have $R={xx}^{⊺}+\sigma^{2}\text{Id}$. Now let $\tilde{x}=\frac{x}{∥x∥}$ and let $v_{1},\ldots ,v_{N-1}$ be an orthonormal basis of ${\left\{x\right\}}^{⊥}$. Letting $A={xx}^{⊺}$, we have $A\tilde{x}=∥x{∥}^{2}\cdot \tilde{x}$, and ${Av}_{i}=0=0\cdot v_{i}$. Hence $\left\{\tilde{x},v_{1},\ldots ,v_{N-1}\right\}$ is an orthonormal basis consisting of eigenvectors of A, corresponding to the eigenvalues $∥x{∥}^{2}$ and 0. Hence $\left\{\tilde{x},v_{1},\ldots ,v_{N-1}\right\}$ is also an orthonormal basis of eigenvectors of $R^{-1}$ corresponding to the eigenvalues $\frac{1}{\sigma^{2}+∥x{∥}^{2}}$ and $\frac{1}{\sigma^{2}}$. Denoting by *p* the orthogonal projection onto the line defined by R·x, this implies for $v\in R^{N_{\text{ch}}}$

(Equation 16) $$R^{-1}v=R^{-1}p\left(v\right)+R^{-1}\left(v-p\left(v\right)\right)=\frac{1}{\sigma^{2}+∥x{∥}^{2}}p\left(v\right)+\frac{1}{\sigma^{2}}\left(v-p\left(v\right)\right)=\frac{1}{\sigma^{2}}v-\frac{1}{\sigma^{2}}\frac{∥x{∥}^{2}}{\sigma^{2}+∥x{∥}^{2}}\frac{{xx}^{⊺}}{∥x{∥}^{2}}v\text{.}$$

This in particular implies

(Equation 17) $$v^{⊺}R^{-1}v=\frac{1}{\sigma^{2}}∥v{∥}^{2}-\frac{1}{\sigma^{2}}\frac{∥x{∥}^{2}}{\sigma^{2}+∥x{∥}^{2}}\frac{{\left(v^{⊺}x\right)}^{2}}{∥x{∥}^{2}}=\frac{∥v{∥}^{2}}{\sigma^{2}}\left(1-\frac{∥x{∥}^{2}}{\sigma^{2}+∥x{∥}^{2}}\cos^{2}\left(x,v\right)\right)\text{,}$$

where cos(x,v) denotes the cosine of the angle between the vectors x and v. These last two equations are also derived in the book of Sekihara and Nagarajan,^28^ section 13.4.

####### Part 2: Proof that the AG and UNG beamformers have no orientation bias

We are now going to prove that AG beamformers and UNG beamformers have no EEG or MEG orientation bias. We start with the EEG case. Let $U=\text{span}\left\{v_{1},\ldots ,v_{N_{\text{ch}}-1}\right\}$ and let $\text{Im}\left(L_{E}\right)$ be the image of the EEG leadfield. Grassmann’s formula (https://proofwiki.org/wiki/Grassmann%27s_Identity) then implies

$$\dim\left(U\cap \text{Im}\left(L_{E}\right)\right)=\dim\left(U\right)+\dim\left(\text{Im}\left(L_{E}\right)\right)-\dim\left(U+\text{Im}\left(L_{E}\right)\right)=\left(N_{\text{ch}}-1\right)+3-N_{\text{ch}}=2\text{.}$$

Hence there exists $\eta_{1},\eta_{2}$ linearly independent with $L_{E}\cdot \eta_{1},L_{E}\cdot \eta_{2}\in U$. Since *U* is the eigenspace of $R^{-1}$ to the eigenvalue $\frac{1}{\sigma^{2}}$, this implies

$$\left(L_{E}^{⊺}R^{-1}L_{E}\right)\cdot \eta_{i}=\frac{1}{\sigma^{2}}\left(L_{E}^{⊺}L_{E}\right)\cdot \eta_{i}\text{,}$$

and

$$\left(L_{E}^{⊺}R^{-2}L_{E}\right)\cdot \eta_{i}=\frac{1}{\sigma^{2}}\left(L_{E}^{⊺}R^{-1}L_{E}\right)\cdot \eta_{i}\text{.}$$

Additionally, since $x=Q\cdot L_{E}\cdot \eta_{\text{sim}}$ is also an eigenvector of $R^{-1}$ corresponding to the eigenvalue $\frac{1}{\sigma^{2}+∥x{∥}^{2}}$, we have

$$\left(L_{E}^{⊺}R^{-1}L_{E}\right)\cdot \eta_{\text{sim}}=\frac{1}{\sigma^{2}+∥x{∥}^{2}}\left(L_{E}^{⊺}L_{E}\right)\cdot \eta_{\text{sim}}\text{,}$$

and

$$\left(L_{E}^{⊺}R^{-2}L_{E}\right)\cdot \eta_{\text{sim}}=\frac{1}{\sigma^{2}+∥x{∥}^{2}}\left(L_{E}^{⊺}R^{-1}L_{E}\right)\cdot \eta_{\text{sim}}\text{.}$$

Hence the smallest generalized eigenvalue of the pairs $\left(L_{E}^{⊺}R^{-1}L_{E},L_{E}^{⊺}L_{E}\right)$ and $\left(L_{E}^{⊺}R^{-2}L_{E},L_{E}^{⊺}R^{-1}L_{E}\right)$ is given by $\frac{1}{\sigma^{2}+∥x{∥}^{2}}$, with a one dimensional eigenspace given by $R\cdot \eta_{\text{sim}}$. Hence the reconstructed orientation for the AG and UNG beamformers in this case is, up to sign, given by $\eta_{\text{sim}}$.

We now continue with the MEG case. Just as in the EEG case, Grassmann’s formula implies that there exists $0\neq \eta \in R^{2}$ so that $L_{M}^{\text{red}}\cdot \eta$ is orthogonal to $L_{M}\cdot \eta_{\text{sim}}$. Again, just as in the EEG case, this implies that η is a generalized eigenvector for the pairs $\left({\left(L_{M}^{\text{red}}\right)}^{⊺}R^{-1}L_{M}^{\text{red}},{\left(L_{M}^{\text{red}}\right)}^{⊺}L_{M}^{\text{red}}\right)$ and $\left({\left(L_{M}^{\text{red}}\right)}^{⊺}R^{-2}L_{M}^{\text{red}},{\left(L_{M}^{\text{red}}\right)}^{⊺}R^{-1}L_{M}^{\text{red}}\right)$ corresponding to the generalized eigenvalue $\frac{1}{\sigma^{2}}$. Computing the remaining eigenvector is slightly more involved than in the EEG case, which is due to the fact that $x=Q\cdot L_{M}\cdot \eta_{\text{sim}}$ is in general not contained in the image of $L_{M}^{\text{red}}$.

Let $\eta_{\text{sim}}^{t}$ be the tangential component and $\eta_{\text{sim}}^{r}$ the radial component of the true orientation $\eta_{\text{sim}}$. Recall that $L_{M}^{\text{red}}=\left(s_{1}\cdot u_{1},s_{2}\cdot u_{2}\right)$. By definition of the singular value decomposition, we know that $u_{3}$ is orthogonal to $u_{1}$ and $u_{2}$, and hence in particular that ${\left(L_{M}^{\text{red}}\right)}^{⊺}\cdot u_{3}=0$. This in particular implies ${\left(L_{M}^{\text{red}}\right)}^{⊺}L_{M}^{\text{red}}\cdot \eta_{\text{sim}}^{t}={\left(L_{M}^{\text{red}}\right)}^{⊺}L_{M}\cdot \eta_{\text{sim}}$. Furthermore, we have $R\cdot u_{3}=\sigma^{2}u_{3}+\left(x^{⊺}u_{3}\right)x$, and hence applying $R^{-1}$ to both sides of this equation yields

(Equation 18) $${\left(L_{M}^{\text{red}}\right)}^{⊺}R^{-1}u_{3}=-\frac{Qx^{⊺}u_{3}}{\sigma^{2}\left(\sigma^{2}+∥x{∥}^{2}\right)}{\left(L_{M}^{\text{red}}\right)}^{⊺}L_{M}^{\text{red}}\eta_{\text{sim}}^{t}\text{.}$$

Noting that $x^{⊺}u_{3}=Qs_{3}\eta_{\text{sim}}^{r}$ and that $L_{M}\cdot \eta_{\text{sim}}=L_{M}^{\text{red}}\eta_{\text{sim}}^{t}+s_{3}\eta_{\text{sim}}^{r}u_{3}$, we can compute

(Equation 19) $$\left({\left(L_{M}^{\text{red}}\right)}^{⊺}R^{-1}L_{M}^{\text{red}}\right)\cdot \eta_{\text{sim}}^{t}=\left(\frac{1}{\sigma^{2}}\cdot \frac{\sigma^{2}+{\left(Q\eta_{\text{sim}}^{r}s_{3}\right)}^{2}}{\sigma^{2}+∥x{∥}^{2}}\right)\left({\left(L_{M}^{\text{red}}\right)}^{⊺}L_{M}^{\text{red}}\right)\cdot \eta_{\text{sim}}^{t}\text{.}$$

Hence $\eta_{\text{sim}}^{t}$ is a generalized eigenvector of the pair $\left({\left(L_{M}^{\text{red}}\right)}^{⊺}R^{-1}L_{M}^{\text{red}},{\left(L_{M}^{\text{red}}\right)}^{⊺}L_{M}^{\text{red}}\right)$ corresponding to the generalized eigenvalue $\frac{1}{\sigma^{2}}\cdot \frac{\sigma^{2}+{\left(Q\eta_{\text{sim}}^{r}s_{3}\right)}^{2}}{\sigma^{2}+∥x{∥}^{2}}<\frac{1}{\sigma^{2}}$. This last inequality follows from ${\left(Q\eta_{\text{sim}}^{r}s_{3}\right)}^{2}={\left(x^{⊺}u_{3}\right)}^{2}<∥x{∥}^{2}$, which itself is a consequence of Bessel’s inequality (see Rudin,^56^ Theorem 4.17) together with the assumption that the true orientation has a non-zero tangential component. Hence the eigenspace corresponding to the smallest generalized eigenvalue is given by $R\cdot \eta_{\text{sim}}^{t}$, and we see that the AG beamformer has no MEG orientation bias.

Noting that $R^{2}u_{3}=\left(\sigma^{2}+\sigma^{2}+∥x{∥}^{2}\right){Ru}_{3}-\sigma^{2}\left(\sigma^{2}+∥x{∥}^{2}\right)u_{3}$, applying $R^{-2}$ to both sides of this equation yields

$${\left(L_{M}^{\text{red}}\right)}^{⊺}R^{-2}u_{3}=\left(\frac{1}{\sigma^{2}}+\frac{1}{\sigma^{2}+∥x{∥}^{2}}\right){\left(L_{M}^{\text{red}}\right)}^{⊺}R^{-1}u_{3}$$

Using this together with $L_{M}\cdot \eta_{\text{sim}}=L_{M}^{\text{red}}\cdot \eta_{\text{sim}}^{t}+s_{3}\eta_{\text{sim}}^{r}u_{3}$ and Equations 18 and 19, we can compute

$$\left({\left(L_{M}^{\text{red}}\right)}^{⊺}R^{-2}L_{M}^{\text{red}}\right)\cdot \eta_{\text{sim}}^{t}=\left(\frac{1}{\sigma^{2}+∥x{∥}^{2}}+\frac{1}{\sigma^{2}}\frac{{\left(Q\eta_{\text{sim}}^{r}s_{3}\right)}^{2}}{\sigma^{2}+{\left(Q\eta_{\text{sim}}^{r}s_{3}\right)}^{2}}\right)\left({\left(L_{M}^{\text{red}}\right)}^{⊺}R^{-1}L_{M}^{\text{red}}\right)\cdot \eta_{\text{sim}}^{t}\text{.}$$

This shows that $\eta_{\text{sim}}^{t}$ is a generalized eigenvector for the pair $\left({\left(L_{M}^{\text{red}}\right)}^{⊺}R^{-2}L_{M}^{\text{red}},{\left(L_{M}^{\text{red}}\right)}^{⊺}R^{-1}L_{M}^{\text{red}}\right)$, and, again using that ${\left(Q\eta_{\text{sim}}^{r}s_{3}\right)}^{2}<∥x{∥}^{2}$, it is straightforward to see that the eigenvalue computed above is smaller than $\frac{1}{\sigma^{2}}$. The eigenspace corresponding to the smallest generalized eigenvalue of the pair $\left({\left(L_{M}^{\text{red}}\right)}^{⊺}R^{-2}L_{M}^{\text{red}},{\left(L_{M}^{\text{red}}\right)}^{⊺}R^{-1}L_{M}^{\text{red}}\right)$ is thus given by $R\cdot \eta_{\text{sim}}^{t}$, which shows that the UNG beamformer has no MEG orientation bias.

####### Part 3: Proof that the UG beamformer has an orientation bias

We now discuss the UG beamformer. In this case, the reconstructed orientation is given by an eigenvector corresponding to the smallest eigenvalue of $L^{⊺}R^{-1}L$, where L is either $L_{E}$ or $L_{M}^{\text{red}}$. It is well known that a vector $\eta_{\text{UG}}$ with $∥\eta_{\text{UG}}∥=1$ is an eigenvector corresponding to the smallest eigenvalue of $L^{⊺}R^{-1}L$ if and only if it is a minimizer of $\eta \mapsto \eta^{⊺}L^{T}R^{-1}L\eta$ over the unit sphere (see e.g. Sekihara and Nagarajan,^28^ section 13.3), and hence

$$\eta_{\text{UG}}=\underset{∥\eta ∥=1}{\arg\;\min}\;\eta^{⊺}L^{⊺}R^{-1}L\eta \overset{\left(17\right)}{=}\underset{∥\eta ∥=1}{\mathrm{argmin}}\;\frac{∥L\eta {∥}^{2}}{\sigma^{2}}\left(1-\frac{∥x{∥}^{2}}{\sigma^{2}+∥x{∥}^{2}}\cos^{2}\left(x,L\eta \right)\right)=\underset{∥\eta ∥=1}{\arg\;\min}\;\underset{=:f_{\sigma }\left(\eta \right)}{\underset{︸}{∥L\eta {∥}^{2}\left(1-\frac{∥x{∥}^{2}}{\sigma^{2}+∥x{∥}^{2}}\cos^{2}\left(x,L\eta \right)\right)}}\text{.}$$

Now define $f_{0}\left(\eta \right)=∥L\eta {∥}^{2}\left(1-\cos^{2}\left(x,L\eta \right)\right)=∥L\eta {∥}^{2}\;\sin^{2}\left(x,L\eta \right)$ and $f_{\infty }\left(\eta \right)=∥L\eta {∥}^{2}$. One can now quickly see that $f_{\sigma }\overset{\sigma \to 0}{\to }f_{0}$ and $f_{\sigma }\overset{\sigma \to \infty }{\to }f_{\infty }$ uniformly over the unit sphere. Since $f_{0}$ and $f_{\infty }$ are continuous, it follows that any cluster point of minimizers of $f_{\sigma }$ as σ→0 is also a minimizer of $f_{0}$, and any cluster point of minimizers of $f_{\sigma }$ as σ→∞ is also a minimizer of $f_{\infty }$.

We first discuss the EEG case. First note that $f_{0}$ is minimized if $\sin^{2}\left(x,L_{E}\cdot \eta \right)=\sin^{2}\left(Q\cdot L_{E}\cdot \eta_{\text{sim}},L_{E}\cdot \eta \right)=0$, which happens if and only if either $\eta =\eta_{\text{sim}}$ or $\eta =-\eta_{\text{sim}}$. Hence ${\arg\;\min}_{∥\eta ∥=1}f_{0}\left(\eta \right)=\left\{\eta_{\text{sim}},-\eta_{\text{sim}}\right\}$, and since the unit sphere is compact this implies that, as σ→0, the UG reconstruction converges up to sign to the true orientation $\eta_{\text{sim}}$.

Furthermore, it is well known that $f_{\infty }\left(\eta \right)=∥L_{E}\cdot \eta {∥}^{2}$ is minimized in the singular vectors of $L_{E}$ corresponding to the smallest singular value. Hence, as σ→∞, it follows that the reconstructed orientations get arbitrarily close to the space of these singular vectors. That the reconstructed orientations actually converge up to sign to the orthogonal projection of the true orientation onto this space (resp. are given by an arbitrary singular vector corresponding to the smallest singular value if this projection is zero) can now be seen by explicitly computing the corresponding eigenvectors using the Bunch-Nielsen-Sorensen formula (see Bunch et al.^57^ for a statement and a proof of this formula).

We now discuss the MEG case. The limiting behavior as σ→∞ can be established in the same way as in the EEG case. In fact, the limiting behavior as σ→0 can also be established in this way, but since the difference between this limit and the desired orientation, namely $\eta_{\text{sim}}^{t}$, is of interest, we want to be more explicit. By Equation 16 we have

$${\left(L_{M}^{\text{red}}\right)}^{⊺}R^{-1}L_{M}^{\text{red}}=\frac{1}{\sigma^{2}}{\left(L_{M}^{\text{red}}\right)}^{⊺}L_{M}^{\text{red}}-\frac{1}{\sigma^{2}}\frac{1}{\sigma^{2}+∥x{∥}^{2}}{\left(L_{M}^{\text{red}}\right)}^{⊺}{xx}^{⊺}L_{M}^{\text{red}}=\frac{1}{\sigma^{2}}\left(\left(\begin{array}{cc} s_{1}^{2} & 0\\ 0 & s_{2}^{2} \end{array}\right)-\frac{Q^{2}}{\sigma^{2}+∥x{∥}^{2}}\left(\begin{array}{c} s_{1}^{2}\cdot \eta_{\text{sim}}^{t_{1}}\\ s_{2}^{2}\cdot \eta_{\text{sim}}^{t_{2}} \end{array}\right)\cdot \left(\begin{array}{cc} s_{1}^{2}\cdot \eta_{\text{sim}}^{t_{1}} & s_{2}^{2}\cdot \eta_{\text{sim}}^{t_{2}} \end{array}\right)\right)$$

If either $\eta_{\text{sim}}^{t_{1}}$ or $\eta_{\text{sim}}^{t_{2}}$ is zero, this matrix is diagonal and the limiting behavior is evident. We shall thus now assume $\eta_{\text{sim}}^{t_{1}},\eta_{\text{sim}}^{t_{2}}\neq 0$. As is shown in Bunch et al.,^57^ the smallest eigenvalue λ of $\sigma^{2}{\left(L_{M}^{\text{red}}\right)}^{⊺}R^{-1}L_{M}^{\text{red}}$ is then given as the unique solution of the equation

$$\frac{{\left(s_{1}^{2}\eta_{\text{sim}}^{t_{1}}\right)}^{2}}{s_{1}^{2}-\lambda }+\frac{{\left(s_{2}^{2}\eta_{\text{sim}}^{t_{2}}\right)}^{2}}{s_{2}^{2}-\lambda }=\alpha \text{,}$$

where $\alpha =\frac{\sigma^{2}+∥x{∥}^{2}}{Q^{2}}$, in the interval $\left(-\infty ,s_{2}^{2}\right)$. Note that the solution λ of this equation in the interval $\left(-\infty ,s_{2}^{2}\right)$ is a strictly increasing function of α∈(0,∞), and furthermore depends continuously on α. Note in particular that $\alpha =\frac{Q^{2}∥L_{M}^{\text{red}}\cdot \eta_{\text{sim}}^{t}{∥}^{2}}{Q^{2}}$ corresponds to λ=0. Hence we have, as σ→0, that $\lambda \left(\frac{\sigma^{2}+∥x{∥}^{2}}{Q^{2}}\right)\to \lambda \left(\frac{∥x{∥}^{2}}{Q^{2}}\right)=:\lambda_{0}$. Note that if the MEG leadfield has a non-vanishing radial component, this implies $\lambda_{0}>\lambda \left(\frac{Q^{2}∥L_{M}^{\text{red}}\cdot \eta_{\text{sim}}^{t}{∥}^{2}}{Q^{2}}\right)=0$. The Bunch-Nielsen-Sorensen formula now finally implies that the eigenvector of ${\left(L_{M}^{\text{red}}\right)}^{⊺}R^{-1}L_{M}^{\text{red}}$ corresponding to the smallest eigenvalue is, up to scaling, given by $\left(\frac{s_{1}^{2}\eta_{\text{sim}}^{t_{1}}}{s_{1}^{2}-\lambda \left(\alpha \right)},\frac{s_{2}^{2}\eta_{\text{sim}}^{t_{2}}}{s_{2}^{2}-\lambda \left(\alpha \right)}\right)$, where $\alpha =\frac{\sigma^{2}+∥x{∥}^{2}}{Q^{2}}$. This implies that the reconstructed orientations converge up to sign to a scalar multiple of $\left(\frac{s_{1}^{2}\eta_{\text{sim}}^{t_{1}}}{s_{1}^{2}-\lambda_{0}},\frac{s_{2}^{2}\eta_{\text{sim}}^{t_{2}}}{s_{2}^{2}-\lambda_{0}}\right)$. If now $s_{1}\neq s_{2}$ and the radial component of the MEG leadfield does not vanish, we see that the limit of the reconstructed orientations is different from $\eta_{\text{sim}}^{t}$.

To summarize, as σ→0, the MEG UG beamformer reconstructions converge up to sign to

$$\tilde{\eta }=c\cdot \left(\begin{array}{c} \frac{s_{1}^{2}\eta_{\text{sim}}^{t_{1}}}{s_{1}^{2}-\lambda_{0}}\\ \frac{s_{2}^{2}\eta_{\text{sim}}^{t_{2}}}{s_{2}^{2}-\lambda_{0}} \end{array}\right)\text{,}$$

where *c* is chosen so that the vector has norm 1, and $\lambda_{0}$ is the unique solution of

$$\frac{{\left(s_{1}^{2}\eta_{\text{sim}}^{t_{1}}\right)}^{2}}{s_{1}^{2}-\lambda }+\frac{{\left(s_{2}^{2}\eta_{\text{sim}}^{t_{2}}\right)}^{2}}{s_{2}^{2}-\lambda }=∥L_{M}\cdot \eta_{\text{sim}}{∥}^{2}$$

on the interval $\left[0,s_{2}^{2}\right)$.

Note that as long as the MEG leadfield is dominated by the tangential part we have $∥L_{M}\cdot \eta_{\text{sim}}{∥}^{2}\approx ∥L_{M}^{\text{red}}\cdot \eta_{\text{sim}}^{t}{∥}^{2}$ and hence $\lambda_{0}\approx 0$, which in turn implies $\tilde{\eta }\approx \tilde{c}\cdot \eta_{\text{sim}}^{t}$, where $\tilde{c}$ is chosen so that $∥\tilde{c}\cdot \eta_{\text{sim}}^{t}∥=1$. As long as the MEG leadfield is dominated by its tangential component we thus expect the UG beamformer for small noise levels to approximately reconstruct the tangential component of the true source orientation.

**Remark.** In the proof above, we have seen that in the generic case, as σ→∞, the UG reconstruction converges up to sign to the singular vector of the lead field corresponding to the smallest singular value. But large noise is not the only reason the UG reconstruction might produce noticeable errors. In fact, if the lead field is badly conditioned, i.e. if the ratio of the largest to the smallest singular value is large, we also expect large errors in the reconstruction. To get an idea of why this is the case, we take a look at the minimization problem defining the UG approach. At the beginning of Part 3 of the proof above, we derived the formula

$$\eta_{\text{UG}}=\underset{∥\eta ∥=1}{\mathrm{argmin}}∥L\eta {∥}^{2}\left(1-\frac{∥x{∥}^{2}}{\sigma^{2}+∥x{∥}^{2}}\cos^{2}\left(x,L\eta \right)\right)\text{.}$$

In the following discussion, we will keep σ fixed. Now the smallest value of ∥Lη∥ for ∥η∥=1 is given by the smallest singular value of L, which is attained in the corresponding singular vector, and similarly, the largest value of ∥Lη∥ for ∥η∥=1 is given by the largest singular value, which is also attained at the corresponding singular vector. Hence a large condition number for L corresponds to a large range of values in the first factor of the above expression, and hence a tendency for the minimizer of the product to be close to the minimizer of the first factor. In the extreme case, when the smallest singular value is zero, we in particular see that the minimizer is given by the singular vector corresponding to the smallest singular value. In total, we thus expect that larger condition numbers of the lead fields result in faster convergence of the UG reconstructions to the singular vectors.

Another expression of this principle can be observed in Figure 4. There, one sees that for the V5 lead field and σ=2.0, the mean error is quite high for orientations close to the largest singular vector $v_{E1}$, while orientations close to the middle singular vector $v_{E2}$ have comparatively low median errors. This is a mathematical necessity. Computing the corresponding expected UG reconstructions using the Bunch-Nielsen-Sorensen formula, one sees that the component of the expected reconstruction in the direction of $v_{E1}$ converges faster to 0 than the component of the expected reconstruction in the direction of $v_{E2}$. When looking at the corresponding expressions, it can be shown that the components in the respective directions are monotonically decreasing functions in the ratios of the singular value in the direction and the smallest singular value. Thus, since the ratio of the largest singular value and the smallest singular value is larger than the ratio of the middle singular value and the smallest singular value, we again see that a larger ratio of singular values leads to a faster convergence against the smallest singular vector.

Finally, we want to discuss a generalization of the statement of theorem 1 for UG beamformers to arbitrary noise covariance matrices.

**Theorem 2.** We now model our signal vector as

$$d\left(t\right)=L\cdot \left(\eta_{\text{sim}}\cdot q\left(t\right)\right)+n\left(t\right)\text{,}$$

where L is a full rank lead field matrix, n(t) is uncorrelated noise with an arbitrary positive definite covariance matrix N, $\eta_{\text{sim}}$ is the true source orientation and q(t) is the source activity. Similar to the derivation of Equation 12, one can then see that the expected covariance matrix R in this case is given by

$$R=N+Q^{2}\left(L\cdot \eta_{\text{sim}}\right){\left(L\cdot \eta_{\text{sim}}\right)}^{⊺}\text{,}$$

where $Q^{2}=\left\langle q{\left(t\right)}^{2}\right\rangle >0$ (see e.g. Moiseev et al.^55^). Now denote by $\eta_{\text{UG}}$ the UG reconstruction using this covariance matrix R. We then have the following generalization of the statement of theorem 1.2.

(1) For ∥N∥→0, the reconstructed orientation $\eta_{\text{UG}}$ converges up to sign to the true orientation $\eta_{\text{sim}}$.
(2) Let τ>0. Let $N=\tau \cdot N_{0}$, where $N_{0}$ is a positive definite matrix so that the smallest eigenvalue of $L^{⊺}N_{0}^{-1}L$ is simple. Then, as τ→∞, the reconstructed orientation $\eta_{\text{UG}}$ converges up to sign to the eigenvector of $L^{⊺}N_{0}^{-1}L$ corresponding to the smallest eigenvalue.

Note that theorem 2.2) is indeed a generalization of the corresponding statement in theorem 1. In that theorem, we have $N=\sigma^{2}\cdot \text{Id}=\sigma^{2}\cdot N_{0}$, with $N_{0}=\text{Id}$. In this case, the smallest eigenvector of $L^{⊺}N_{0}^{-1}L=L^{⊺}L$ is simply the smallest singular vector of L.

Additionally note that based on theorem 2.2), and the argument used to derive it, we can more generally expect that, in the case that the noise is much stronger than the signal, the orientation $\eta_{UG}$ will be close to the eigenvector of $L^{⊺}N^{-1}L$ corresponding to the smallest eigenvalue.

*Proof.* In order to keep the presentation concise, we will restrict ourselves to a sketch of the proof.

Using the Sherman–Morrison formula, we have

$$L^{⊺}R^{-1}L=L^{⊺}\left(N^{-1}-\frac{Q^{2}N^{-1}\cdot \left(L\eta_{\text{sim}}\right)\cdot {\left(L\eta_{\text{sim}}\right)}^{⊺}\cdot N^{-1}}{1+Q^{2}\cdot \left\langle L\eta_{\text{sim}},N^{-1}\cdot L\eta_{\text{sim}}\right\rangle }\right)L=L^{⊺}N^{-1}L-\frac{\left(L^{⊺}N^{-1}L\right)\cdot \left(Q\eta_{\text{sim}}\right)\cdot {\left(Q\eta_{\text{sim}}\right)}^{⊺}\cdot \left(L^{⊺}N^{-1}L\right)}{1+\left\langle Q\eta_{\text{sim}},L^{⊺}N^{-1}LQ\eta_{\text{sim}}\right\rangle }={\left({\left(L^{⊺}N^{-1}L\right)}^{-1}+Q^{2}\eta_{\text{sim}}\cdot \eta_{\text{sim}}^{⊺}\right)}^{-1}\text{,}$$

where the Sherman–Morrison formula was applied in the first and the last line. We thus see that

$$\eta_{\text{UG}}=\nu_{\min}\left\{L^{⊺}R^{-1}L\right\}=\nu_{\max}\left\{{\left(L^{⊺}N^{-1}L\right)}^{-1}+Q^{2}\cdot \eta_{\text{sim}}\cdot \eta_{\text{sim}}^{⊺}\right\}\text{.}$$

Using standard results from spectral theory, one can see that ∥N∥→0 implies $∥{\left(L^{⊺}N^{-1}L\right)}^{-1}∥\to 0$. Together with standard results from the perturbation theory of the hermitian eigenproblem, as they can e.g. be found in the book of Saad,^58^ 3.2.2, one can then see that as ∥N∥→0, the UG reconstruction converges up to sign to the eigenvector corresponding to the largest eigenvalue of $Q^{2}\cdot \eta_{\text{sim}}\cdot \eta_{\text{sim}}^{⊺}$, which is given by the true orientation $\eta_{\text{sim}}$. This shows 1).

Statement 2) can be derived analogously, where in this case we interpret $Q^{2}\cdot \eta_{\text{sim}}\cdot \eta_{\text{sim}}^{⊺}$ as a perturbartion of ${\left(L^{⊺}N^{-1}L\right)}^{-1}$. Then essentially the same reasoning as for statement 1) shows that $\eta_{\text{UG}}$ converges up to sign to $\nu_{\max}\left\{{\left(L^{⊺}N_{0}^{-1}L\right)}^{-1}\right\}$, which is given by the eigenvector corresponding to the smallest eigenvalue of $L^{⊺}N_{0}^{-1}L$.
