# Supplementary material for: Comparing the performance of beamformer algorithms in estimating orientations of neural sources
Source: iScience. 2024 Feb 6;27(3):109150. doi: 10.1016/j.isci.2024.109150 (PMC10901088; doi:10.1016/j.isci.2024.109150)
Supplement: Document S1. Figures S1–S6 and Tables S1–S4 [file mmc1.pdf]

**iScience, Volume *27***

## Supplemental information

# Comparing the performance of beamformer algorithms in estimating orientations of neural sources

**Yvonne Buschermöhle, Malte B. Höltershinken, Tim Erdbrügger, Jan-Ole Radecke, Andreas Sprenger, Till R. Schneider, Rebekka Lencer, Joachim Gross, and Carsten H. Wolters**

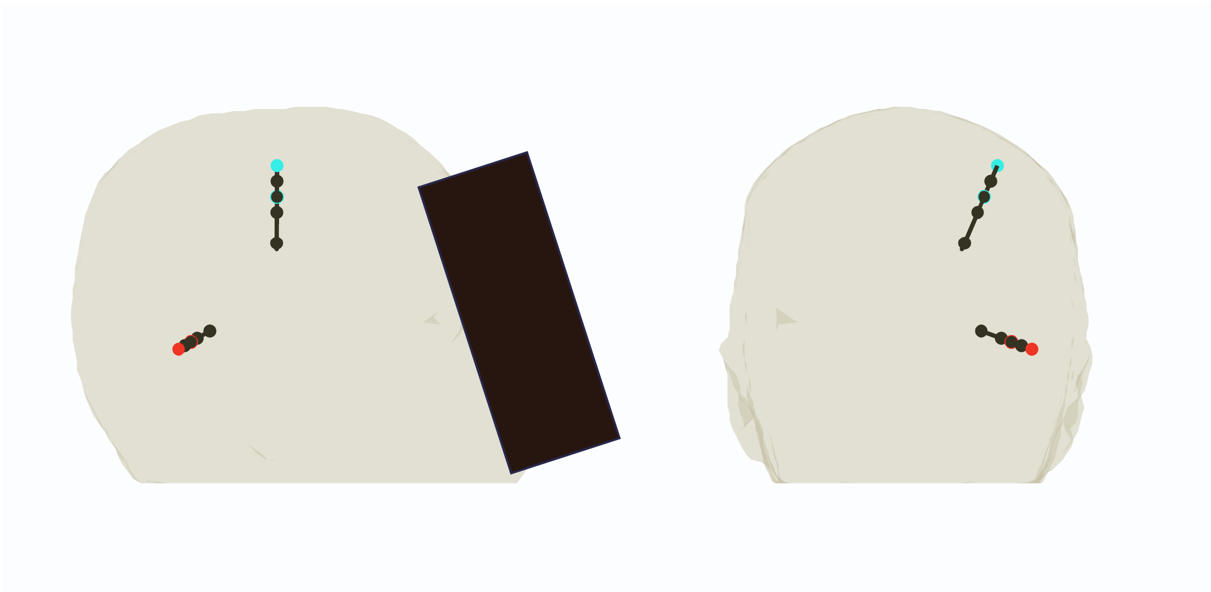

**Figure S1: Locations of targets in different depths, related to STAR Methods.**
Targets differing in depth defined from the closest skull point of targets V5 and FEF. Colored markers show closest skull points (red: closest skull point V5; blue: closest skull point FEF) and black markers color-edged are the original targets V5 (red) and FEF (blue). Black markers show the chosen targets for different source depths.

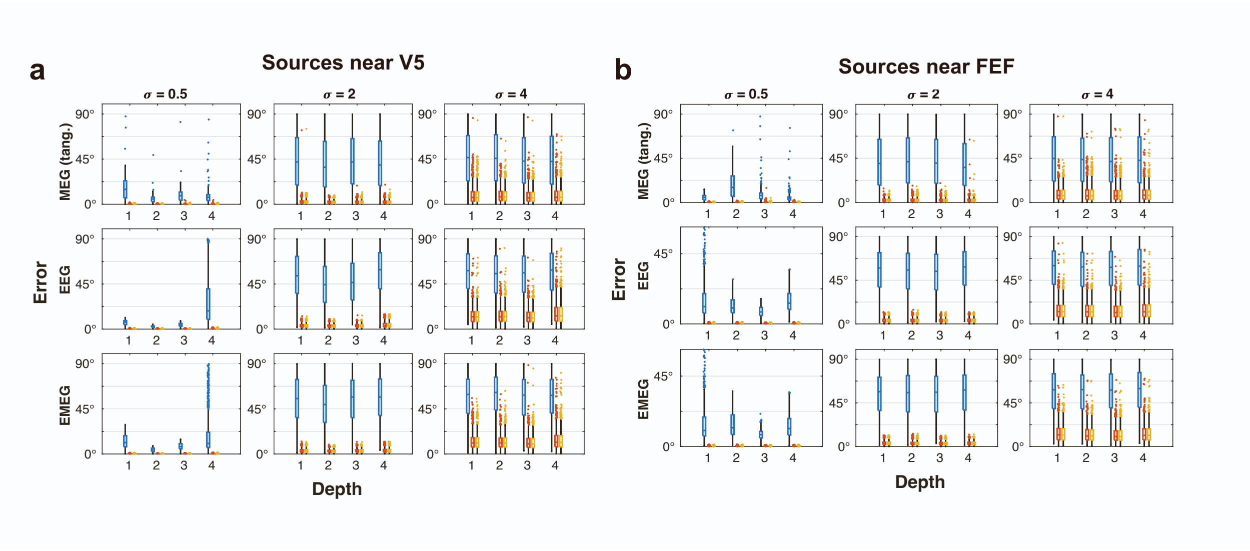


**Figure S2: Estimation errors for different orientations in all beamformers (UG, UNG, AG) and modalities (MEG, EEG, EMEG) for targets of varying depths, related to Figure 1.**
Angles between estimate and ground truth for 1000 random orientations per condition in targets close to V5 (a) and FEF (b) for different beamformers (UG: blue; UNG: orange; AG: yellow). Targets differ in depths with 1: closest to the skull surface and 4: deepest target location. Conditions differ in noise levels ($\sigma = [0.5, 2, 4]$) and modalities (MEG, EEG, combined EMEG). For MEG, the estimation error is evaluated in the tangential plane only. Signal strength is equal in all scenarios.

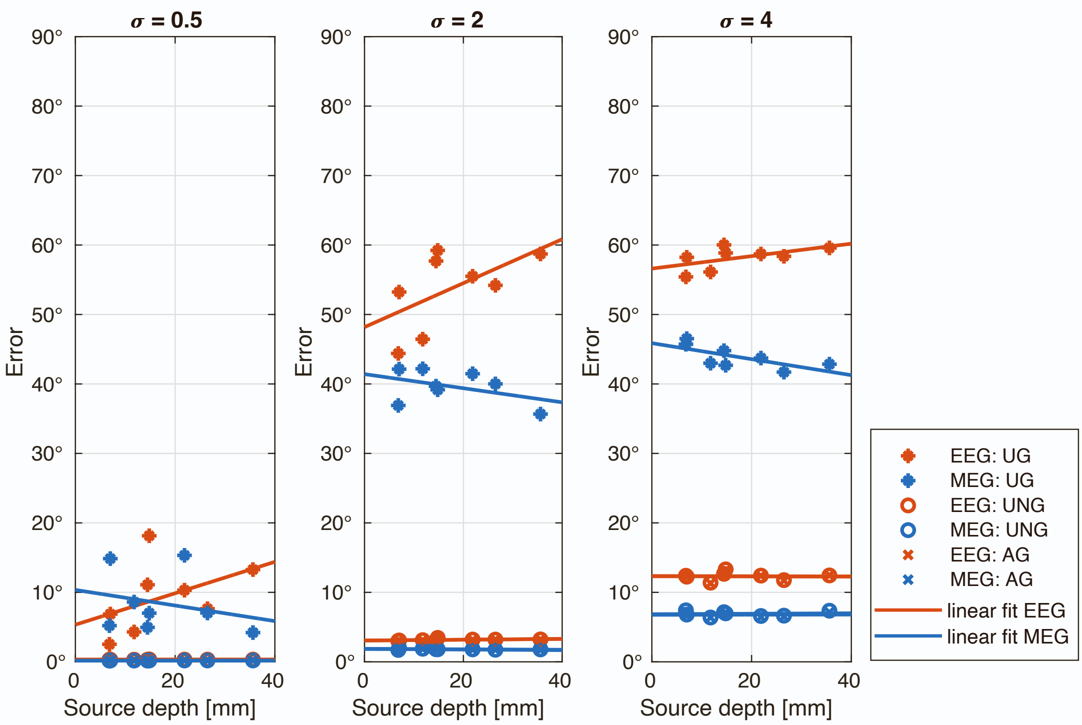


**Figure S3: Dependency of median estimation error on source depth, related to Figure 1.** Dependency of the median estimation error on the source depth. Conditions differ in noise level ($\sigma = [0.5, 2, 4]$), beamformer (UG, UNG and AG) and modality (MEG: blue; EEG: orange). Linear least square fits are included in the figures for all conditions.

**Table S1: Correlation between source depth and median estimation error, related to Figure 1.** Pearson's R values (r) and corresponding significance values (p) for the correlation between source depth and median estimation error. Conditions differ in modality (EEG and MEG) and beamformer choice (UG, UNG and AG). Significance values p<0.05 (uncorrected for multiple comparison) are highlighted.

| Beamformer | Noise Level | EEG | | MEG | |
|---|---|---|---|---|---|
| | | r | p | r | p |
| UG | 1 | 0.449 | 0.264 | -0.257 | 0.538 |
| | 2 | 0.572 | 0.139 | -0.426 | 0.292 |
| | 3 | 0.559 | 0.149 | -0.695 | 0.056 |
| UNG | 1 | 0.065 | 0.878 | 0.696 | 0.055 |
| | 2 | 0.424 | 0.295 | -0.387 | 0.343 |
| | 3 | -0.024 | 0.955 | 0.088 | 0.837 |
| AG | 1 | 0.073 | 0.863 | 0.411 | 0.312 |
| | 2 | 0.424 | 0.296 | -0.595 | 0.120 |
| | 3 | -0.019 | 0.964 | 0.027 | 0.949 |

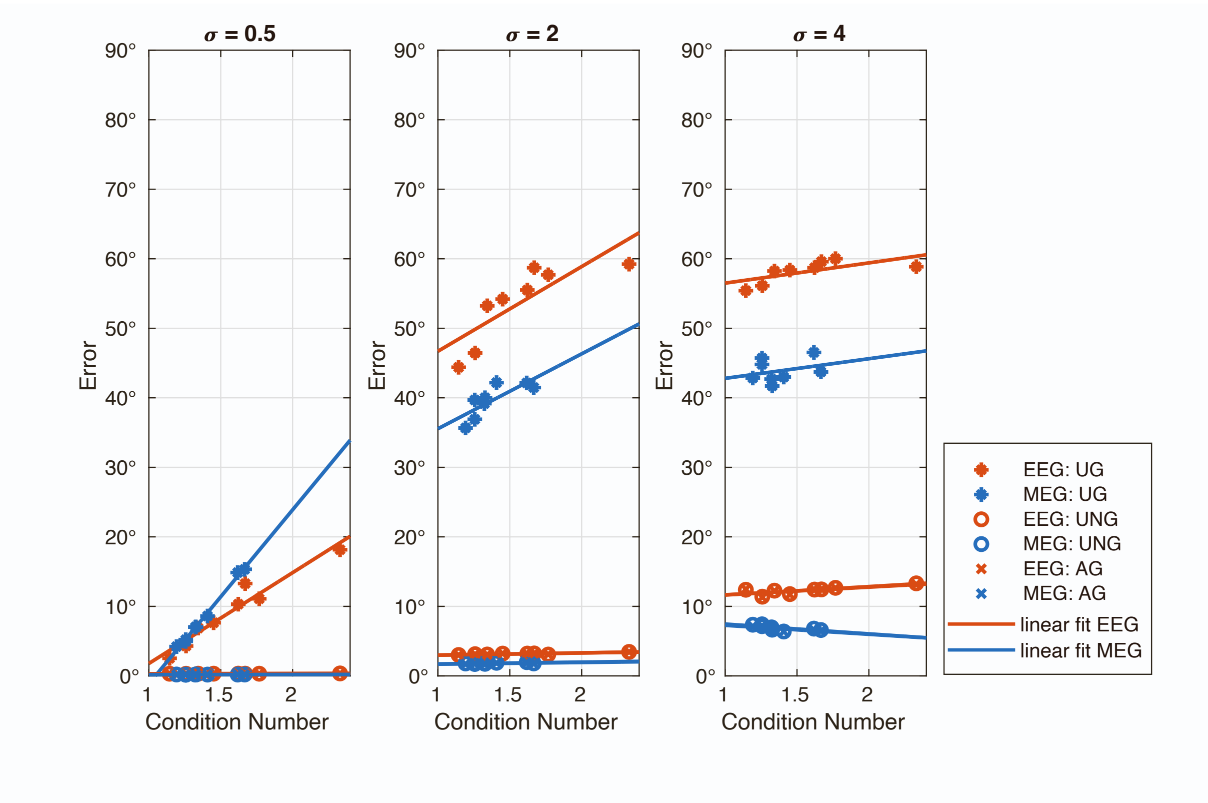


**Figure S4: Dependency of median estimation error on condition number, related to Figure 1.** Dependency of the median estimation error on the condition number. Conditions differ in noise level ($\sigma = [0.5, 2, 4]$), beamformer (UG, UNG and AG) and modality (MEG: blue; EEG: orange). Linear least square fits are included in the figures for all conditions.

**Table S2: Correlation between condition number and median estimation error, related to Figure 1.**

Pearson's R values (r) and corresponding significance values (p) for the correlation between Condition Number and median estimation error. Conditions differ in modality (EEG and MEG) and beamformer choice (UG, UNG and AG). Significance values $p<0.05$ (uncorrected for multiple comparison) are highlighted.

| Beamformer | Noise Level | EEG | | MEG | |
|---|---|---|---|---|---|
| | | r | p | r | p |
| UG | 1 | 0.967 | **<0.001** | 0.996 | **<0.001** |
| | 2 | 0.820 | **0.013** | 0.786 | **0.021** |
| | 3 | 0.676 | 0.067 | 0.300 | 0.477 |
| UNG | 1 | 0.873 | **0.005** | 0.333 | 0.420 |
| | 2 | 0.843 | **0.009** | 0.554 | 0.154 |
| | 3 | 0.786 | **0.021** | -0.654 | 0.079 |
| AG | 1 | 0.863 | **0.006** | 0.512 | 0.195 |
| | 2 | 0.888 | **0.003** | 0.563 | 0.150 |
| | 3 | 0.725 | **0.042** | -0.681 | 0.063 |

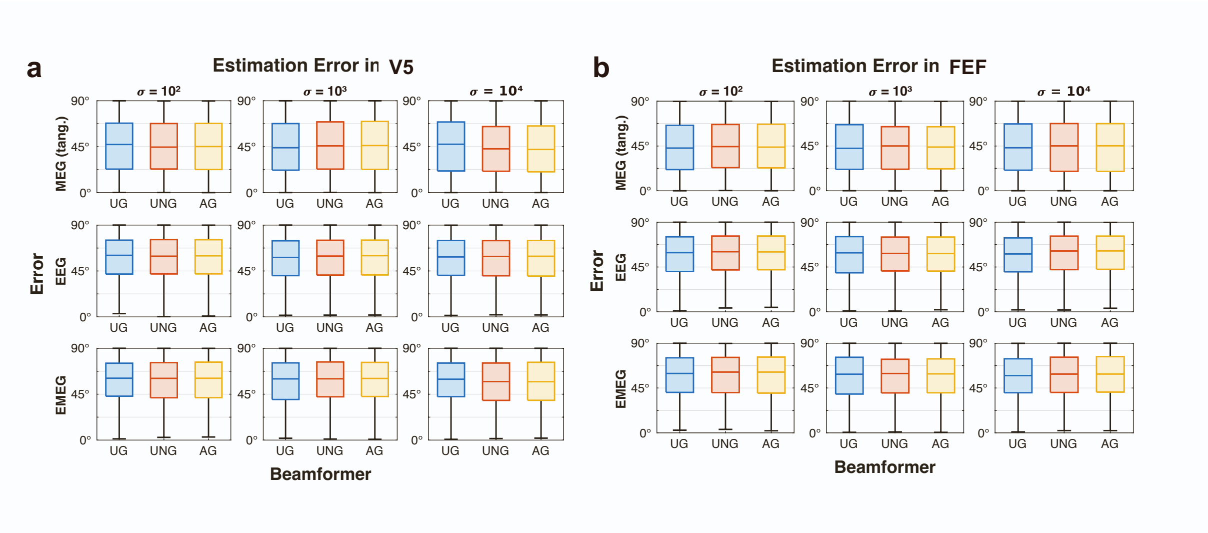


**Figure S5: Estimation errors for different orientations in all beamformers (UG, UNG, AG) and modalities (MEG, EEG, EMEG), related to Figure 1.**
Angles between estimate and ground truth for 1000 random orientations per condition in V5 (a) and FEF (b) for different beamformers (UG: blue; UNG: orange; AG: yellow). Conditions differ in noise levels ($\sigma = [10^2, 10^3, 10^4]$) and modalities (MEG, EEG, combined EMEG). For MEG, the estimation error is evaluated in the tangential plane only. Signal strength is equal in all scenarios.

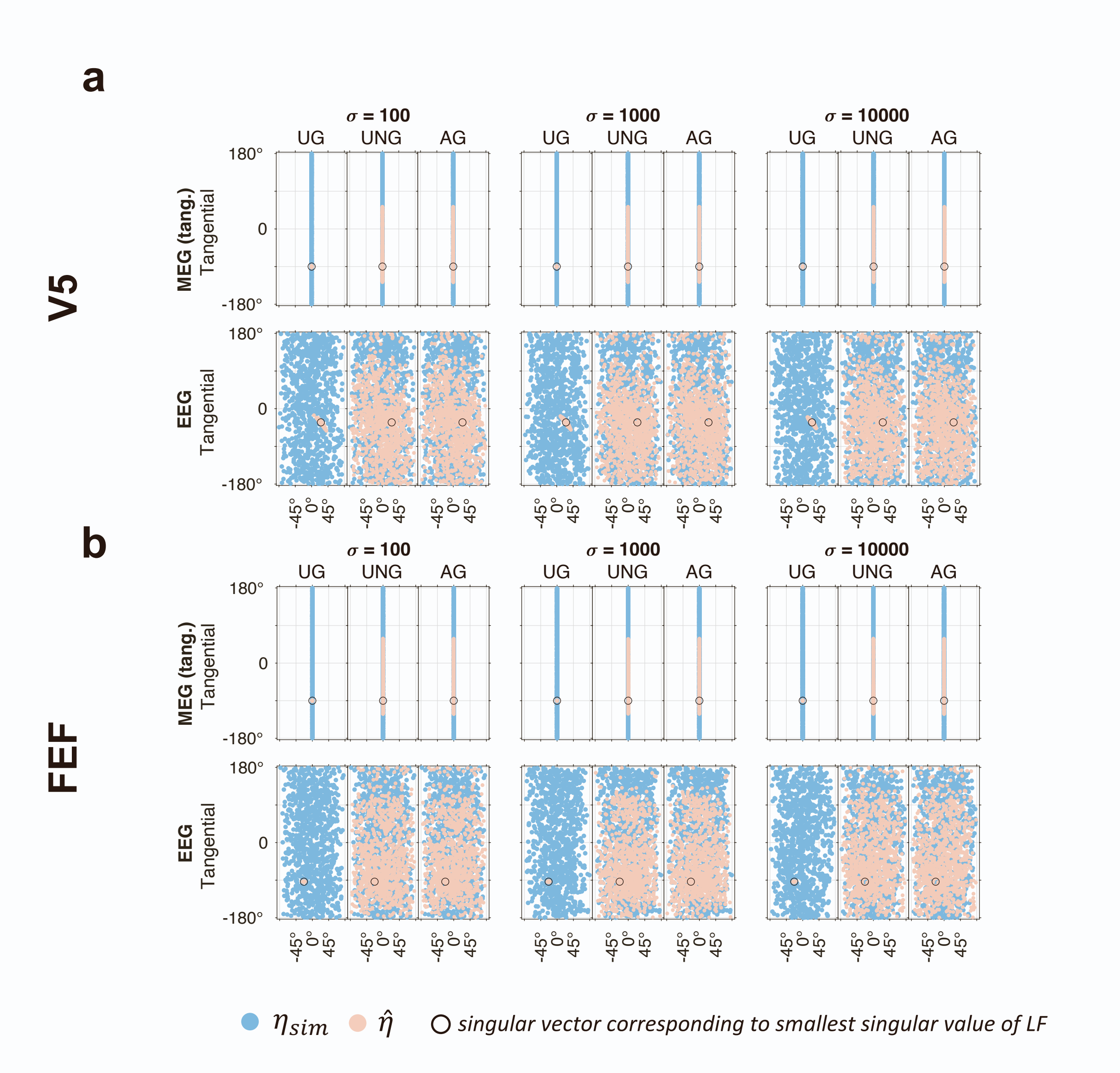


**Figure S6: Difference between sets of ground truth orientations and estimated orientations, related to Figure 2.**

Contrasting the set of simulated orientations $\eta_{\text{sim}}$ (blue) and the reconstructed orientations $\hat{\eta}$ (orange) for varying modalities (MEG and EEG) and noise levels $\sigma$ in both targets V5 (a) and FEF (b). Coordinates represent the orientations in spherical coordinates (x-axis: elevation/ radial component; y-axis: azimuth/ tangential component). While simulated orientations (blue) cover the entire sphere, UG reconstructions converge towards a fixed value with increasing noise levels in both targets and modalities. The convergence value is the singular vector corresponding to the smallest singular value of the leadfield (MEG or EEG). In UNG and AG, estimates cover (at least) half the sphere of possible orientations. Since beamformers only reconstruct up to a difference of 180°, this is equivalent to covering the entire sphere of possible orientations and points to no convergence value. Signal strength is equal in all scenarios.

**Table S3: Confidence intervals of orientation estimation errors for varying noise levels in V5, related to Figure 3.**

95% confidence intervals of the mean orientation estimation value in V5 for varying noise levels in EEG ($\sigma_E$ between 0.5 and 10), while the noise level in MEG is kept constant ($\sigma_M$ = 4).

FEF: Frontal Eye Field; UG: Unit-Gain; UNG: Unit-Noise-Gain; AG: Array-Gain; MEG: Magnetoencephalography; EEG: Electroencephalography; EMEG: combined EEG and MEG; $\sigma_E$**:** Noise level in EEG; $\sigma_M$**:** Noise level in MEG

| | **V5** | | | | | |
|---|---|---|---|---|---|---|
| $\sigma_E$ | **UG** | | **UNG** | | **AG** | |
| | **EEG** | **EMEG** | **EEG** | **EMEG** | **EEG** | **EMEG** |
| **0.5** | [3.15; 3.35] | [34.20; 37.10] | [0.32; 0.34] | [6.65; 7.51] | [0.32; 0.34] | [6.64; 7.50] |
| **1** | [12.11; 13.07] | [37.16; 39.92] | [0.90; 0.97] | [6.73; 7.52] | [0.90; 0.96] | [6.66; 7.43] |
| **1.5** | [22.44; 24.72] | [46.39; 49.07] | [1.84; 1.96] | [6.42; 7.16] | [1.84; 1.96] | [6.35; 7.07] |
| **2** | [30.04; 32.65] | [50.10; 52.75] | [3.14; 3.36] | [7.36; 8.20] | [3.14; 3.35] | [7.35; 8.17] |
| **3** | [42.61; 45.41] | [52.56; 55.42] | [7.03; 7.52] | [8.97; 9.73] | [7.01; 7.50] | [8.89; 9.63] |
| **4** | [48.29; 51.11] | [53.94; 56.66] | [12.78; 13.84] | [11.59; 12.54] | [12.74; 13.80] | [11.51; 12.46] |
| **5** | [52,16; 54.91] | [55.15; 57.84] | [21.09; 23.00] | [16.77; 18.41] | [21.07; 22.98] | [16.76; 18.41] |
| **6** | [52.23; 54.99] | [54.45; 57.22] | [28.85; 31.24] | [22.23; 24.44] | [28.83; 31.23] | [22.09; 24.29] |
| **7** | [52.66; 55.44] | [54.96; 57.63] | [35.28; 37.96] | [26.23; 28.72] | [35.27; 37.95] | [26.18; 28.68] |
| **8** | [54.69 ;57.39] | [55.71; 58.38] | [40.33 ;43.12] | [29.93; 32.69] | [40.34; 43.12] | [29.77; 32.52] |
| **9** | [53.80; 56.52] | [54.84; 57.60] | [43.00; 45.79] | [32.04; 34.87] | [42.97; 45.75] | [31.87; 34.70] |
| **10** | [53.69; 56.45] | [55.23; 57.90] | [45.70; 48.55] | [33.59; 36.48] | [45.70; 48.55] | [33.46; 36.35] |

**Table S4: Confidence intervals of orientation estimation errors for varying noise levels in FEF, related to Figure 3.**

95% confidence intervals of the mean orientation estimation value in FEF for varying noise levels in EEG ($\sigma_E$ between 0.5 and 10), while the noise level in MEG is kept constant ($\sigma_M$ = 4).

FEF: Frontal Eye Field; UG: Unit-Gain; UNG: Unit-Noise-Gain; AG: Array-Gain; MEG: Magnetoencephalography; EEG: Electroencephalography; EMEG: combined EEG and MEG; $\sigma_E$**:** Noise level in EEG; $\sigma_M$**:** Noise level in MEG

| | **FEF** | | | | | |
|---|---|---|---|---|---|---|
| $\sigma_E$ | **UG** | | **UNG** | | **AG** | |
| | **EEG** | **EMEG** | **EEG** | **EMEG** | **EEG** | **EMEG** |
| **0.5** | [9.18; 9.84] | [32.18; 34.92] | [0.34; 0.37] | [6.74; 7.57] | [0.34; 0.36] | [6.68; 7.50] |
| **1** | [36.40; 39.22] | [41.64; 44.49] | [0.97; 1.05] | [6.77; 7.71] | [0.97; 1.04] | [6.65; 7.57] |
| **1.5** | [47.22; 50.14] | [49.21; 52.08] | [1.93; 2.08] | [6.94; 7.77] | [1.93; 2.08] | [6.86; 7.70] |
| **2** | [51.22; 54.04] | [52.11; 54.89] | [3.31; 3.55] | [7.54; 8.34] | [3.30; 3.54] | [7.49; 8.27] |
| **3** | [53.57; 56.38] | [53.76; 56.55] | [7.41; 7.97] | [9.36; 10.27] | [7.39; 7.95] | [9.25; 10.15] |
| **4** | [53.70; 56.47] | [53.71; 56.49] | [13.32; 14.46] | [12.19; 13.37] | [13.27; 14.40] | [12.19; 13.27] |
| **5** | [54.75; 57.46] | [54.57; 57.28] | [21.86; 23.87] | [18.05; 19.81] | [21.86; 23.87] | [17.97; 19.71] |
| **6** | [54.96; 57.70] | [54.75; 57.50] | [29.11; 31.53] | [22.63; 24.73] | [29.09; 31.51] | [22.57; 24.68] |
| **7** | [54.41; 57.15] | [54.21; 56.97] | [36.23; 38.99] | [27.52; 30.16] | [36.20; 38.96] | [27.38; 30.02] |
| **8** | [55.16; 57.85] | [54.85; 57.56] | [40.87; 43.69] | [31.26; 33.97] | [40.80; 43.63] | [31.04; 33.74] |
| **9** | [55.22; 57.95] | [54.94; 57.68] | [42.90; 45.70] | [32.40; 35.07] | [42.93; 45.72] | [32.36; 35.03] |
| **10** | [54.58; 57.32] | [54.34; 58.09] | [46.13; 49.00] | [34.84; 37.81] | [46.08; 48.94] | [34.67; 37.64] |
